# Rapid discrimination of geographical origin and analysis of chemical characterization of tobacco leaves from multiple countries

**DOI:** 10.3389/fchem.2026.1721371

**Published:** 2026-03-20

**Authors:** Ranran Kou, Cong Wang, Ran Wan, Mingliang Su, Heng Xu, Yufeng Fu, Yun Lin, XinHua Song, Yiming Bi, Le Zhao, Junwei Guo, Hongbo Wang, Zechun Liu, Song Yang, Cong Nie

**Affiliations:** 1 Key Laboratory of Tobacco Chemistry, Zhengzhou Tobacco Research Institute of China National Tobacco Corporation (CNTC), Zhengzhou, China; 2 Technology Center, China Tobacco Fujian Industrial Co., Ltd., Xiamen, China; 3 Technology Center, China Tobacco Henan Industrial Co., Ltd., Zhengzhou, China; 4 Technology Center, China Tobacco Guangdong Industrial Co., Ltd., Guangzhou, China; 5 Technology Center, China Tobacco Shandong Industrial Co., Ltd., Jinan, China; 6 Technology Center, China Tobacco Zhejiang Industrial Co., Ltd., Hangzhou, China

**Keywords:** chemical composition, chemical feature interpretation, chemometrics, geographical origin traceability of tobacco, permutation feature importance, support vector machine with hybrid kernel

## Abstract

Tobacco is a globally cultivated crop featuring distinct quality variations among leaves from different geographical origins. To develop a rapid, robust, and accurate method for multi-origin traceability, this study employed near-infrared spectroscopy combined with rapid chemical composition analysis to obtain 70 chemical components in samples from nine major tobacco-producing regions in China and four other countries (the United States, Brazil, Zimbabwe, and Zambia). One-way analysis of variance (ANOVA) and hierarchical cluster analysis (HCA) were used to investigate regional chemical differences. Discrimination models were built using a support vector machine (SVM), a backpropagation neural network, and a random forest. The best model was interpreted using permutation feature importance (PFI) to identify key markers for origin discrimination. One-way ANOVA revealed significant differences (p ≤ 0.001), and HCA demonstrated clear regional patterns. The SVM-hybrid kernel achieved the best performance with 97.96% test accuracy and macro-average recall, precision, and F1 scores of 0.9836, 0.9806, and 0.9821, respectively. The PFI algorithm was employed to identify and rank the top 20 key chemical components influencing the geographical origin discrimination. The top ten key components were Fru-Asn, succinic acid, rutin, Fru-Val, sulfate, serine, phosphate, starch, potassium, and Fru-Gly. This study integrated chemometrics, near-infrared, rapid chemical analysis, and interpretable machine learning to accurately distinguish tobacco origins, reveal regional traits, and offer insights into geographical traceability and chemical profiling.

## 1 Introduction

Tobacco is a globally cultivated economic crop with substantial scientific and commercial value (Bareschino et al., 2021; Cui L. et al., 2023; Cui L.-L. et al., 2023; Wu et al., 2013). Owing to the influence of various environmental factors, such as soil type and climate, tobacco leaves from different regions exhibit notable differences in chemical composition and overall quality (Ma et al., 2019). Recently, issues such as counterfeiting, illicit circulation, and tobacco smuggling have increasingly worsened. In certain supply chains, low-quality or unidentified tobacco is falsely labeled as originating from premium regions to obtain higher market returns (Binette et al., 2009; Nguyen et al., 2020). Therefore, establishing rapid, effective, and reliable methods for tracing the geographic origin of tobacco is crucial for combating fraud and ensuring quality control.

Current traceability methods fall into two main categories: instrumental signal- and composition-based approaches. Instrumental signaling methods include near-infrared (NIR) spectroscopy, electronic noses, and thermal analysis. NIR spectroscopy requires simple sample pretreatment and nondestructive sampling and exhibits fast analysis speed, good repeatability, and reproducibility. In addition, it has been widely used in recent years (Richter et al., 2019; Wang et al., 2018a; Wu et al., 2020; Xiao et al., 2020). Its combination with machine learning techniques has been extensively applied to tobacco origin identification. Wang and Yang (2023) proposed a generalized learning system for the Takagi (TS) fuzzy subsystem based on NIR spectroscopy for the rapid identification of tobacco origin. This study used tobacco leaf samples collected from eight different regions in the Guizhou Province to achieve a prediction accuracy of 95.59%. Xiang et al. (2020) identified the geographical origin and grade of flue-cured tobacco based on NIR spectroscopy. Xue et al. (2015) developed regional classification models for tobacco from Guangdong, Fujian, Chongqing, and Sichuan using electronic nose data and constellation clustering. Zhang et al. (2020) established an aroma-type classification model based on the thermal analysis of tobacco from eight Chinese aroma types, optimized using a genetic-algorithm-based support vector machine (SVM).

Chemical-composition-based traceability methods involve the use of isotopes, volatile metabolites, and chemical components. Cui L. et al. (2023) conducted traceability studies at both provincial and municipal levels based on stable isotopes and elemental content. Cui L.-L. et al. (2023) further analyzed 260 tobacco samples from six Chinese provinces using isotope ratio mass spectrometry and inductively coupled plasma mass spectrometry, followed by orthogonal partial least squares-discriminant analysis and random forest (RF) analysis. Zhang et al. (2013) utilized gas chromatography–mass spectrometry to analyze volatile metabolites for regional discrimination. Xie et al. (2008) used Mahalanobis distance based on the total sugar, reducing sugar, nicotine, total nitrogen, potassium, and chlorine content to classify tobacco origins.

However, traceability methods based on instrument signals offer a limited capability to reveal the chemical characteristics of tobacco leaves. Chemical-composition-based approaches often rely on traditional laboratory techniques that involve complex pretreatments, long detection cycles, and high resource consumption. Furthermore, research based on these two methods has mostly focused on a few typical regions with limited sample coverage. A rapid traceability model covering tobacco leaves from multiple countries has not yet been established, making it difficult to comprehensively determine the differences in chemical characteristics among tobacco leaves of different origins. SVM excels in handling high-dimensional data, such as the 70 chemical components analyzed here, and small-to-moderate sample sizes, as its kernel function can efficiently map nonlinear relationships between chemical features and geographic origins without overfitting, which is critical for distinguishing subtle chemical differences among closely related regions. A BPNN, with its multilayered neural network structure, can capture complex interactive relationships between multiple chemical components and origin labels. RF, an ensemble learning method, offers strong robustness to outliers and measurement noise in chemical component detection, ensuring stable model performance even when faced with slight variations in sample analysis results. This stability is further enhanced by optimizing the hyperparameters of all three models using particle swarm optimization (PSO), which helps each algorithm adapt to the specific distribution of the tobacco chemical dataset. Permutation feature importance (PFI) is a model-agnostic method that assesses the importance of each feature by randomly permuting its values to disrupt the relationship between that feature and the output variable and then measuring the resulting change in model prediction performance. If permuting a feature significantly degrades the model performance, the feature is considered important; conversely, if the model performance remains largely unaffected, the feature has little influence (Abdulrashid et al., 2025; Schwarz et al., 2024). In this study, the PFI was applied to interpret the optimal classification model, identify the key chemical constituents that contribute to the discrimination of tobacco origins, and rank their relative importance.

In this study, NIR spectroscopy combined with rapid chemical composition analysis technology (Guo et al., 2023; Kou et al., 2025; Li et al., 2025; Liang et al., 2022) was employed to obtain data on 70 chemical components from tobacco samples collected across 13 regions. These components largely covered the major and semi-micro chemical constituents of tobacco leaves. The samples included those from nine major tobacco-producing provinces in China (Yunnan, Sichuan, Guizhou, Chongqing, Henan, Hunan, Fujian, Shandong, and Heilongjiang) and four other countries (the United States, Brazil, Zimbabwe, and Zambia). One-way analysis of variance (ANOVA) and hierarchical cluster analysis (HCA) were used to evaluate the interregional differences in chemical composition. Three machine learning models—SVM, backpropagation neural network (BPNN), and RF—were employed to construct high-accuracy classification models. The best-performing model was further interpreted using the PFI to identify the key chemical markers contributing to origin discrimination. These findings provide valuable insights into the traceability, authenticity, and chemical characteristics of agricultural products.

## 2 Materials and methods

### 2.1 Materials

A total of 1,717 tobacco leaf samples were collected, all of which were flue-cured leaves obtained from enterprise storage warehouses after mellowing. The specific tobacco varieties were consistent with the mainstream cultivars grown in each producing region to ensure representativeness of the samples. The samples originated from nine major tobacco-producing provinces in China—Yunnan, Sichuan, Guizhou, Chongqing, Henan, Hunan, Fujian, Shandong, and Heilongjiang—which broadly represent the principal tobacco cultivation regions of the country. Additionally, samples were obtained from four other countries: the United States, Brazil, Zimbabwe, and Zambia, all of which are among the world’s major tobacco-producing countries. Mixed-grade tobacco leaves were used for these international sources, and no specific regional (state/province level) labels were available. Detailed information on the sample distribution and classification is presented in Table 1.

**TABLE 1:** Overview of tobacco leaf sample information.

No.	Country	Province	Number of samples
1	China	Yunnan	360
2	China	Sichuan	103
3	China	Guizhou	157
4	China	Chongqing	92
5	China	Henan	206
6	China	Hunan	103
7	China	Fujian	124
8	China	Shandong	24
9	China	Heilongjiang	100
10	The United States	—	64
11	Brazil	—	129
12	Zimbabwe	—	154
13	Zambia	—	101
Total			1717

### 2.2 Chemical composition analysis

In previous studies, we established and validated a chemical composition prediction model (Guo et al., 2023; Kou et al., 2025; Li et al., 2025; Liang et al., 2022) based on NIR spectra using the JIT-PLS algorithm. The predicted chemical components included the common chemical components of tobacco leaves, cations and anions, polyphenols, polyacids, higher fatty acids, amino acids, and Amadori compounds. The model exhibited good predictive performance for most chemical components. Except for glycine, cystine, Fru-Amb, and Fru-Phe, which showed relatively poorer prediction accuracy, satisfactory predictive performance was achieved for the remaining components (Liang et al., 2022), enabling the conclusions to reliably reflect the true chemical differences among tobacco leaves from different geographical origins. These components encompass both major and trace substances found in tobacco leaves and represent a crucial foundation for tobacco quality (Table 2). The modeling process is discussed in the following section.

**TABLE 2:** Chemical components (70) in tobacco leaves.

No.	Type	Compound name	Number of chemical components
1	Routine chemical components	Total alkaloids, Reducing sugar, Total sugar, Total nitrogen, Starch	5
2	Cations and anions	Potassium, Chlorine, Sulfate, Phosphate, Magnesium, Calcium	6
3	Polyphenols	Neo-chlorogenic acid, Chlorogenic acid, Cryptochlorogenic acid, Scopoletin, Rutin	5
4	Polyacids and higher fatty acids	Oxalic acid, Malonic acid, Succinic acid, Malic acid, Citric acid, Vanillic acid, Myristic acid, Palmitic acid, Linoleic acid, Oleic acid + Linolenic acid, Stearic acid, Arachidic acid	12
5	Amino acids	Aspartic acid, Threonine, Serine, Asparagine, Glutamic acid, Glutamine, Glycine, Alanine, Valine, Cystine, Methionine, Isoleucine, Leucine, Tyrosine, Phenylalanine, 4-Aminobutyric acid (GABA), Lysine, Histidine, Tryptophan, Arginine, Proline	21
6	Amadori compounds	N-(1-Deoxy-d-glucose-1-yl) Ammonia (Glu-An), N-(1-deoxy-D-fructos-1-yl) aminobutyric (Fru-Amb), N-(1-deoxy-D-fructos-1-yl) Histidine (Fru-His), N-(1-deoxy-D-fructos-1-yl) Proline (Fru-Pro), N-(1-deoxy-D-fructos-1-yl) Valine (Fru-Val), N-(1-deoxy-D-fructos-1-yl) Threonine (Fru-Thr), N-(1-deoxy-D-fructos-1-yl) Glycine (Fru-Gly), N-(1-deoxy-D-fructos-1-yl) Alanine (Fru-Ala), N-(1-deoxy-D-fructos-1-yl) Asparagine (Fru-Asn), N-(1-deoxy-D-fructos-1-yl) Asparticacid (Fru-Asp), N-(1-deoxy-D-fructos-1-yl) Glutarnine (Fru-Gln), N-(1-deoxy-D-fructos-1-yl) Glutamicacid (Fru-Glu), N-(1-deoxy-D-fructos-1-yl) Isoleucine (Fru-Ile), N-(1-deoxy-D-fructos-1-yl) Leucine (Fru-Leu), N-(1-deoxy-D-fructos-1-yl) Tyrosine (Fru-Tyr), N-(1-deoxy-D-fructos-1-yl) Phenylalanine (Fru-Phe), N-(1-deoxy-D-fructos-1-yl) Tryptophan (Fru-Trp)	17
7	Others	pH, Dichloromethane extract, Solanesol, Neo-phytene	4
Total			70

Tobacco leaves were obtained from enterprise stockpiles of mellowed, flue-cured leaves, followed by sample preparation and spectral acquisition. The procedure for sample preparation and spectral acquisition was as follows: First, the tobacco leaf samples were placed in an FD240 oven (Binder GmbH, Germany) and dried at 40 °C until the moisture content reached 6%–8%. They were then pulverized using a ZM200 grinder (Retsch GmbH, Germany) and passed through a 60-mesh (0.25 mm) sieve to prepare a homogeneous tobacco powder for analysis. Spectral acquisition was performed using an Antaris Fourier Transform NIR (FT-NIR) spectrometer (Thermo Nicolet, United States), equipped with an integrating sphere diffuse reflectance sampling system, an InGaAs detector, Result spectral acquisition software, TQ Analyst 6.2 intelligent analysis software, and a sample cup (Φ 4.8 cm). The analysis was conducted in an environment with a temperature range of 20 °C–27 °C and a relative humidity of 30%–50%. Samples were loaded into the sample cup (filling height > 10 mm) and pressed flat with a 280 g tamper before being placed on the instrument’s scanning stage. The instrument’s scanning range was set to 10,000–4,000 cm^-1^ with a resolution of 8 cm^-1^, and each collected spectrum consisted of 1,557 data points. During the measurement, the powdered samples were loaded into a spinning sample cup to enhance sampling representativeness. Each sample was measured three times, and the similarities between the three spectra were calculated. When the similarity reached 99.99%, the average of the three spectra was considered as the final spectrum for that sample. To improve the signal-to-noise ratio (SNR), each measurement was considered as an average of 64 scans. Throughout this process, a background spectrum was collected every 20 min to correct and eliminate interference from environmental and instrumental drift.

The Kennard–Stone algorithm was used to divide the sample set into calibration and validation subsets. The chemical compositions of the samples were determined using various analytical methods (see Liang et al., 2022; Supplementary Table S1 for details). Prior to modeling, the spectra were preprocessed using multiplicative scatter correction (MSC) and a first-order derivative after Savitzky-Golay smoothing. The chemical composition prediction models were constructed using the JIT-PLS algorithm based on the NIR spectrum (Guo et al., 2023; Li et al., 2025; Liang et al., 2022). The results indicated that the model achieved very high accuracy in predicting 70 chemical components. All computations were performed using MATLAB.

In this study, the chemical composition data of 1,717 tobacco leaf samples were obtained using the model described above.

### 2.3 Statistical analysis

One-way ANOVA followed by *post hoc* Tukey’s HSD tests were performed using SPSS software (version 27.0, IBM, United States) to evaluate significant differences among the tobacco leaf samples. HCA was conducted using Multi Experiment Viewer software (MeV, version 4.9.0).

### 2.4 Traceability model construction

**FIGURE 1:**
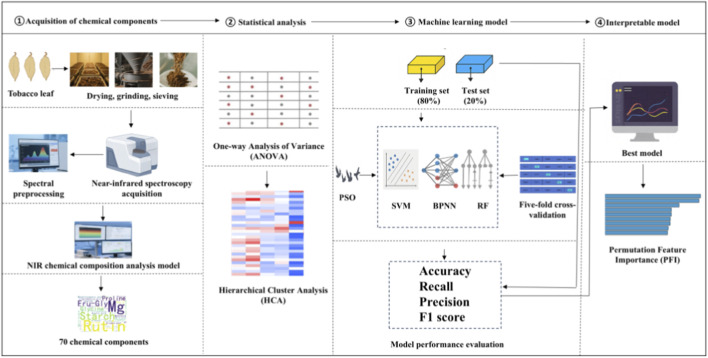
Methodological flowchart.

A flowchart of the methodology is shown in Figure 1. This study employed NIR spectroscopy combined with rapid chemical composition analysis to obtain 70 chemical components from samples collected from nine major tobacco-producing regions of China and four other countries (the United States, Brazil, Zimbabwe, and Zambia). One-way ANOVA and HCA were used to investigate regional chemical differences. Discrimination models were built using SVM, BPNN, and RF. The best model was interpreted using PFI to identify key markers for origin discrimination.

#### 2.4.1 Five-fold cross-validation and external validation

Stratified sampling was used to divide the tobacco samples into a training set (80%) and an independent test set (20%) without overlapping data. Fivefold cross-validation was applied to the training set to determine the optimal parameters, whereas the independent test set was excluded from the cross-validation process. After optimizing each model and identifying the best parameters, the entire training set (80%) was used to retrain the model, and the independent test set (20%) was used for the final performance evaluation.

#### 2.4.2 Modeling methods

To establish reliable models for the classification of tobacco leaf origin, three machine learning algorithms were employed: SVM, BPNN, and RF. To enhance the classification performance of each model, PSO was used to search for an optimal set of hyperparameters.

**FIGURE 2:**
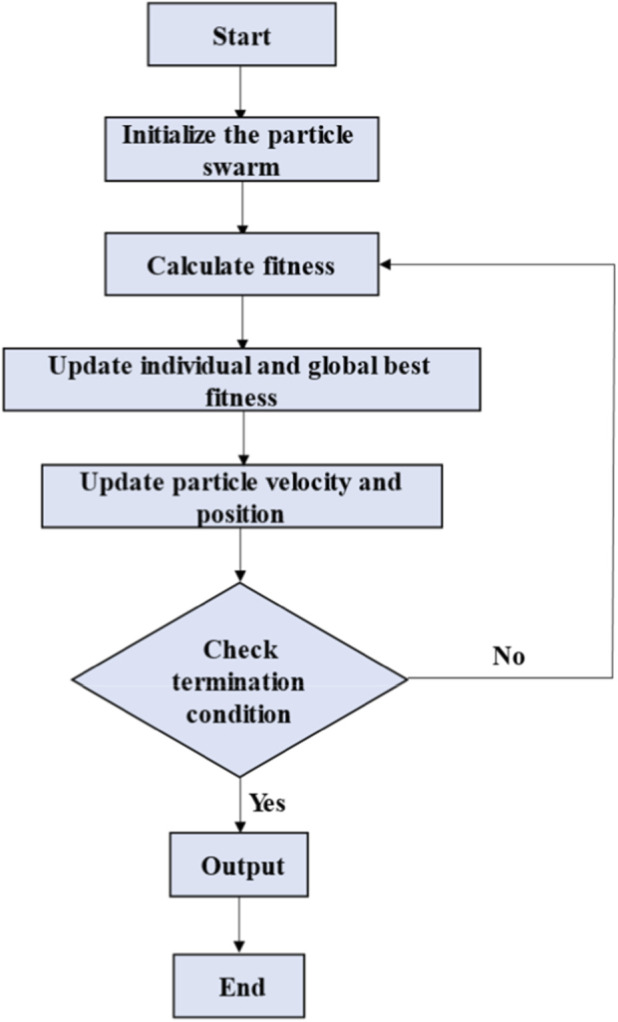
Workflow of the particle swarm optimization (PSO) algorithm.

PSO is a population-based stochastic optimization algorithm inspired by the collective intelligence behavior observed in biological populations, such as bird flocks and fish schools. Owing to its fast convergence, strong global search capability, and adaptability, PSO has been widely applied in machine learning optimization tasks (Li et al., 2024; Wang et al., 2018b; Xu et al., 2024; Zhu et al., 2024). The workflow of the PSO algorithm is illustrated in Figure 2. The process begins by initializing the particle swarm, in which each particle is assigned a random position and velocity in the search space. Next, the fitness of each particle is evaluated using the objective function. The algorithm then updates the individual and global best-fitness values, tracking each particle’s personal best position and the best position determined by the entire swarm. Based on these values, the particle velocities and positions are adjusted to guide the swarm toward the optimal solutions. A termination condition that can reach the maximum number of iterations or achieve a satisfactory fitness value is then checked. If the condition is not satisfied (No), the algorithm loops back to recalculate the fitness and continues to update the particles. If the condition is satisfied (yes), the algorithm outputs the global best solution and terminates the process. This iterative process allows the swarm to converge to an optimal or near-optimal solution over successive iterations.

The inertia weight in the PSO algorithm was set to 0.9 (linearly decayed to 0.4). The acceleration constants *c*1 and *c*2 were both 1.5, and the random factors *r*1 and *r*2 were randomly generated in the range [0, 1]. Specifically, the inertia weight was set to 0.9, and it linearly decreased to 0.4 to balance global exploration in the early stages of the algorithm with local exploitation in the later stages. The acceleration constants were both set to 1.5, allowing particles to maintain a proper balance when approaching their personal best positions (cognitive learning) and the global best position (social learning), thereby preventing overly fast convergence or excessive jumps. Random factors are uniformly generated in the range of [0, 1] to introduce search randomness and enhance the algorithm’s ability to escape local optima.

The average accuracy of the fivefold cross-validation was used as the fitness function and applied to the parameter optimization of the SVM, BPNN, and RF models. In the PSO algorithm of the SVM model, the influence of different kernel functions and their parameter settings on classification performance is emphasized. The parameter settings of each kernel function are listed in Table 3, and the parameter ranges of PSO are listed in Table 4. In the PSO algorithm of the BPNN model, key parameters, such as the number of hidden layers, the number of neurons in each layer, and the learning rate, were adjusted. The tuning range of PSO is listed in Table 4. In the PSO algorithm of the RF model, key parameters, such as the number of trees and the minimum number of samples required for each leaf node, are adjusted. The ranges of PSO parameters are listed in Table 4.

**TABLE 3:** Parameter settings for the SVM kernel function.

Kernel function	Parameter setting
Linear kernel	$y=x_{1}\cdot {x_{2}}^{\prime }$
Polynomial kernel	$y={\left(c_{1}\cdot x_{1}\cdot {x_{2}}^{\prime }+c_{2}\right)}^{c_{3}}$
Gaussian kernel	$y=\exp\left(-\frac{{\left(x_{1}-x_{2}\right)\left(x_{1}-x_{2}\right)}^{\prime }}{\sigma^{2}}\right)$
Sigmoid kernel	$y=\tanh\left(a{\cdot x}_{1}\cdot {x_{2}}^{\prime }+c\right)$
Hybrid kernel	$y_{0}=x_{1}\cdot {x_{2}}^{\prime }$
	$y_{1}={\left(c_{1}\cdot x_{1}\cdot {x_{2}}^{\prime }+c_{2}\right)}^{c_{3}}$
	$y_{2}=\exp\left(-\frac{{\left(x_{1}-x_{2}\right)\left(x_{1}-x_{2}\right)}^{\prime }}{\sigma^{2}}\right)$ $y=my_{0}+ny_{1}+qy_{2}$

**TABLE 4:** Particle swarm optimization tuning range.

Model	Tuning range
SVM- linear kernel	—
SVM- polynomial kernel	c_1_: [1/80, 1/40]; *c*_2_: [0.1, 3]; *c*_3_: [2, 4]
SVM- Gaussian kernel	*σ* ^*2*^: [10, 50]
SVM-sigmoid kernel	*a*: [1/100, 1]; *c*: [-5, 5]
SVM- hybrid kernel	c_1_: [1/80, 1/40]; *c*_2_: [0.1, 3]; *c*_3_: [2, 4]
	*σ* ^*2*^: [10, 50]
	*m*,*n*,*q*: [0, 1]; *m* + *n* + *q* = 1
BPNN	The number of hidden layers: [1, 5]
	The number of neurons in each layer: [2, 40]
	The learning rate: [0.001, 0.1]
RF	The number of trees: [50, 200]
	The minimum number of samples required for each leaf node: [2, 10]

#### 2.4.3 Modeling performance evaluation

Accuracy (*Acc*), recall (*R*), precision (*P*), F1 score (F1), macro-average recall (*macro-R*), macro-average precision (*macro-P*), macro-average F1 score (*macro-*F1), and other indicators were selected to evaluate the model performance. Accuracy reflects the overall correctness of the model, recall measures the model’s ability to identify positive samples, and precision indicates the reliability of the positive predictions. The F1 score balances precision and recall. For multiclass classification, macro-average metrics, macro-average recall, macro-average precision, and macro-average F1 score determine the arithmetic or harmonic mean across all classes, ensuring a fair assessment of each class. These metrics are crucial for evaluating model performance because they capture different aspects of classification quality. Accuracy provides an overall measure but can be misleading in imbalanced datasets. Precision and recall reflect the reliability and sensitivity of positive predictions, respectively. The F1 score offers a balanced assessment, particularly when false positives and false negatives are significant. Macro-average metrics ensure that minority classes are fairly evaluated, thus providing a comprehensive reflection of the model’s classification ability. The calculation formulae are presented in Equations 1–7.

$$Acc=\frac{\sum_{i=1}^{n}TP_{i}}{\sum_{i=1}^{n}\left(TP_{i}+FP_{i}\right)}$$ (1)

$$R=\frac{TP}{TP+FN}$$ (2)

$$P=\frac{TP}{TP+FP}$$ (3)

$$F1=\frac{2\times P\times R}{P+R}$$ (4)

$$macro-R=\frac{1}{n}\sum_{i=1}^{n}\frac{TP_{i}}{TP_{i}+FN_{i}}$$ (5)

$$macro-P=\frac{1}{n}\sum_{i=1}^{n}\frac{TP_{i}}{TP_{i}+FP_{i}}$$ (6)

$$macro-F1=\frac{2\times macro_{-}R\times macro_{-}P}{macro_{-}R+macro_{-}P}$$ (7)

where *TP* represents the number of samples that were actually positive and predicted as positive, *FN* represents the number of samples that were actually positive but predicted as negative, and *FP* represents the number of samples that were actually negative but predicted as positive.

#### 2.4.4 Chemical feature importance analysis

The PFI is a model-agnostic method that assesses the importance of each feature by randomly permuting its values to disrupt the relationship between that feature and the output variable and then measuring the resulting change in model prediction performance. If permuting a feature significantly degrades the model performance, the feature is considered important; conversely, if the model performance remains largely unaffected, the feature has little influence (Abdulrashid et al., 2025; Schwarz et al., 2024). In this study, the PFI was applied to interpret the optimal classification model, identify the key chemical constituents that contribute to the discrimination of tobacco origins, and rank their relative importance.

## 3 Results and discussion

### 3.1 Chemical composition differences of tobacco leaves from different origins

#### 3.1.1 Results of one-way ANOVA

One-way ANOVA, followed by the *post hoc* Tukey’s HSD test, was conducted on tobacco leaf samples. Table 5 lists the mean contents and standard deviations of the 70 chemical components in tobacco leaves from the five countries. Except for glycine, cystine, Fru-Amb, and Fru-Phe, the predictive performance of the models for the remaining chemical components was satisfactory (Liang et al., 2022), enabling the derived conclusions to reliably reflect the true chemical differences among tobacco leaves from different geographical origins. The results indicated that, except for chlorine, significant differences (p-value ≤ 0.001) were observed in chemical composition among tobacco leaves from these countries.

**TABLE 5:** Mean contents ± standard deviations of 70 chemical components in tobacco leaf samples from different countries.

Chemical component	China	United States	Brazil	Zimbabwe	Zambia	Significance level
	n = 1269	n = 64	n = 129	n = 154	n = 101	
Total alkaloids (%)	2.50 ± 0.60a	3.02 ± 0.23b	3.98 ± 0.39c	3.13 ± 0.38b	2.68 ± 0.33d	***
Reducing sugar (%)	20.96 ± 4.09a	11.69 ± 2.62b	12.02 ± 1.52b	16.63 ± 2.29c	16.69 ± 2.40c	***
Total sugar (%)	23.45 ± 5.27a	12.33 ± 3.21b	12.70 ± 1.95b	19.35 ± 3.10c	18.47 ± 2.82c	***
Total nitrogen (%)	2.19 ± 0.31a	2.41 ± 0.19b	2.65 ± 0.17c	2.08 ± 0.17d	2.09 ± 0.19d	***
Potassium (%)	1.96 ± 0.50a	2.38 ± 0.19b	2.02 ± 0.16a	2.38 ± 0.20b	2.68 ± 0.16c	***
Chlorine (%)	0.48 ± 0.39a	0.58 ± 0.12a	0.47 ± 0.10a	0.44 ± 0.18a	0.43 ± 0.13a	ns
pH	5.19 ± 0.16a	5.03 ± 0.12bd	5.02 ± 0.09b	5.08 ± 0.13cd	5.04 ± 0.09bc	***
Starch (%)	4.41 ± 1.20ae	4.71 ± 0.87be	3.17 ± 0.49c	4.04 ± 0.59d	4.20 ± 0.64ad	***
Dichloromethane extract (%)	4.26 ± 0.82a	6.28 ± 0.82b	5.42 ± 0.42c	4.52 ± 0.46d	4.14 ± 0.51a	***
Solanesol (mg/g)	8.76 ± 3.72a	15.87 ± 3.53b	14.11 ± 2.32c	9.95 ± 2.10d	8.16 ± 2.35a	***
Sulfate (mg/g)	8.94 ± 4.21a	9.30 ± 1.31a	5.94 ± 1.08b	5.12 ± 1.14b	4.91 ± 1.16b	***
Phosphate (mg/g)	4.16 ± 0.47a	5.38 ± 0.40be	4.90 ± 0.30c	5.38 ± 0.40de	5.23 ± 0.35b	***
Magnesium (%)	0.45 ± 0.13a	0.56 ± 0.06b	0.61 ± 0.06c	0.53 ± 0.06be	0.51 ± 0.04de	***
Calcium (%)	2.22 ± 0.64a	1.46 ± 0.22b	1.70 ± 0.17c	1.90 ± 0.22d	1.64 ± 0.24bc	***
Neo-chlorogenic acid (mg/g)	1.30 ± 0.27a	0.89 ± 0.15b	1.36 ± 0.16ad	1.45 ± 0.23c	1.41 ± 0.28cd	***
Chlorogenic acid (mg/g)	9.21 ± 1.84a	5.82 ± 1.30b	8.61 ± 1.17c	9.81 ± 1.37d	9.10 ± 1.62ac	***
Cryptochlorogenic acid (mg/g)	1.92 ± 0.35a	1.36 ± 0.13b	2.04 ± 0.21c	2.29 ± 0.31d	2.24 ± 0.38d	***
Scopoletin (mg/g)	0.24 ± 0.08a	0.48 ± 0.08b	0.38 ± 0.05c	0.28 ± 0.07d	0.32 ± 0.06e	***
Rutin (mg/g)	8.11 ± 2.21a	5.25 ± 1.82b	8.60 ± 1.30a	9.38 ± 1.75c	8.59 ± 1.50a	***
Oxalic acid (mg/g)	11.44 ± 2.33a	12.20 ± 1.31b	12.67 ± 1.12bd	13.29 ± 1.24cd	11.26 ± 1.26a	***
Malonic acid (mg/g)	1.45 ± 0.48a	1.76 ± 0.56b	1.89 ± 0.49b	1.25 ± 0.37c	1.08 ± 0.35d	***
Succinic acid (mg/g)	0.27 ± 0.04a	0.39 ± 0.04b	0.36 ± 0.04c	0.39 ± 0.04b	0.41 ± 0.04d	***
Malic acid (mg/g)	51.14 ± 16.41a	39.01 ± 5.34b	52.87 ± 5.32ad	57.55 ± 5.64cd	53.89 ± 4.57ac	***
Citric acid (mg/g)	6.37 ± 2.09a	6.24 ± 1.01a	7.44 ± 0.99b	8.07 ± 1.19c	8.09 ± 1.62bc	***
Vanillic acid (mg/g)	0.13 ± 0.02a	0.15 ± 0.01b	0.16 ± 0.01c	0.13 ± 0.01a	0.13 ± 0.01a	***
Myristic acid (mg/g)	0.15 ± 0.02a	0.19 ± 0.02b	0.17 ± 0.01c	0.15 ± 0.01a	0.14 ± 0.01d	***
Palmitic acid (mg/g)	2.85 ± 0.15a	2.78 ± 0.07b	2.67 ± 0.09c	2.67 ± 0.11c	2.76 ± 0.13b	***
Linoleic acid (mg/g)	1.72 ± 0.25a	2.20 ± 0.19b	2.07 ± 0.10c	1.87 ± 0.09d	1.85 ± 0.12d	***
Oleic acid + Linolenic acid (mg/g)	3.60 ± 0.44a	3.65 ± 0.29ad	3.50 ± 0.21bd	3.32 ± 0.24c	3.44 ± 0.34bc	***
Stearic acid (mg/g)	0.57 ± 0.04a	0.55 ± 0.02b	0.51 ± 0.03c	0.50 ± 0.03c	0.51 ± 0.04c	***
Arachidic acid (mg/g)	0.13 ± 0.01a	0.15 ± 0.01b	0.14 ± 0.01c	0.13 ± 0.01a	0.13 ± 0.01a	***
Aspartic acid (μg/g)	281.70 ± 102.36a	484.22 ± 119.21b	460.61 ± 69.57b	290.94 ± 83.59ad	323.89 ± 67.00cd	***
Threonine (μg/g)	44.27 ± 29.20a	41.32 ± 27.21a	64.90 ± 22.72b	27.46 ± 17.39c	26.75 ± 15.69c	***
Serine (μg/g)	149.03 ± 92.36a	82.51 ± 87.73b	144.52 ± 72.91a	110.19 ± 76.17b	84.37 ± 53.62b	***
Asparagine (μg/g)	1145.01 ± 846.41a	2141.25 ± 868.02b	2204.86 ± 606.44b	1094.49 ± 541.48a	938.55 ± 502.07a	***
Glutamic acid (μg/g)	144.29 ± 92.79a	196.72 ± 97.37b	263.15 ± 74.66c	130.45 ± 62.88ae	118.91 ± 53.07de	***
Glutamine (μg/g)	437.87 ± 392.49a	217.61 ± 313.55b	399.83 ± 258.08a	212.29 ± 212.97b	129.82 ± 144.69b	***
Glycine (μg/g)	26.18 ± 9.62a	37.87 ± 13.20b	38.51 ± 7.87b	24.76 ± 8.25ad	22.99 ± 7.97cd	***
Alanine (μg/g)	293.15 ± 113.50a	322.16 ± 124.02ad	364.98 ± 88.31bd	231.63 ± 78.83c	199.09 ± 76.57c	***
Valine (μg/g)	299.23 ± 60.63a	234.41 ± 58.35b	260.15 ± 42.96c	219.27 ± 32.24b	229.66 ± 35.81b	***
Cystine (μg/g)	87.70 ± 10.27ad	95.53 ± 8.28b	90.07 ± 6.39a	85.55 ± 5.94cd	89.57 ± 6.40a	***
Methionine (μg/g)	12.44 ± 3.10a	16.94 ± 2.28b	16.68 ± 2.22b	10.25 ± 2.20c	11.17 ± 2.21c	***
Isoleucine (μg/g)	9.86 ± 2.41a	10.65 ± 2.08b	9.89 ± 1.30ab	6.50 ± 1.71c	8.31 ± 1.45d	***
Leucine (μg/g)	17.09 ± 4.41a	20.16 ± 3.09b	21.49 ± 2.72b	16.59 ± 2.35a	17.00 ± 2.12a	***
Tyrosine (μg/g)	54.59 ± 16.64a	58.06 ± 11.85a	70.92 ± 9.14b	46.72 ± 10.94c	52.21 ± 9.34a	***
Phenylalanine (μg/g)	145.95 ± 71.81a	165.31 ± 60.49a	200.80 ± 48.98b	101.74 ± 43.78c	101.10 ± 36.91c	***
4-Aminobutyric acid (GABA) (μg/g)	101.59 ± 53.81a	140.79 ± 57.00b	158.91 ± 46.32b	72.79 ± 33.65c	68.02 ± 31.51c	***
Lysine (μg/g)	24.45 ± 15.98a	27.44 ± 16.05a	36.56 ± 12.72b	13.22 ± 7.95c	13.25 ± 6.55c	***
Histidine (μg/g)	104.70 ± 62.70a	104.32 ± 54.14a	154.81 ± 46.59b	75.33 ± 38.02c	64.84 ± 32.11c	***
Tryptophan (μg/g)	100.59 ± 59.88a	83.45 ± 54.90ade	141.22 ± 49.28b	71.54 ± 40.21cd	64.85 ± 32.56ce	***
Arginine (μg/g)	34.44 ± 14.15a	41.23 ± 13.77b	47.59 ± 10.13c	23.21 ± 9.15d	23.21 ± 8.37d	***
Proline (μg/g)	6628.86 ± 2787.12a	3174.83 ± 1913.41b	5044.72 ± 1565.00c	3590.15 ± 1337.04b	2856.99 ± 1111.62b	***
Glu-An (μg/g)	224.43 ± 117.40a	256.91 ± 105.28a	392.23 ± 89.46b	185.49 ± 81.87c	158.85 ± 75.88c	***
Fru-Amb (μg/g)	2212.78 ± 365.80a	1629.76 ± 207.60b	1694.78 ± 201.92b	1622.66 ± 161.80b	1706.48 ± 227.45b	***
Fru-His (μg/g)	67.78 ± 34.29a	28.91 ± 23.43b	45.71 ± 22.08c	32.29 ± 17.53b	24.02 ± 14.66b	***
Fru-Pro (μg/g)	9223.37 ± 2485.89a	4790.33 ± 2084.25b	6291.02 ± 1039.45c	7425.29 ± 1132.19d	7318.31 ± 1301.53d	***
Fru-Val (μg/g)	210.48 ± 43.13a	140.01 ± 21.55b	174.05 ± 26.92c	164.14 ± 29.93c	194.44 ± 39.54d	***
Fru-Thr (μg/g)	16.12 ± 4.33a	11.00 ± 1.93b	12.99 ± 2.53c	11.70 ± 2.02b	12.12 ± 2.33bc	***
Fru-Gly (μg/g)	26.65 ± 4.65a	29.31 ± 3.37b	30.10 ± 2.98b	27.25 ± 2.60a	29.61 ± 2.92b	***
Fru-Ala (μg/g)	2343.18 ± 336.82a	1881.22 ± 261.87b	1906.53 ± 134.67b	2038.02 ± 168.43c	2098.05 ± 199.14c	***
Fru-Asn (μg/g)	3742.73 ± 991.49a	3292.33 ± 961.02b	3321.28 ± 557.40b	2784.13 ± 617.59c	2836.78 ± 568.90c	***
Fru-Asp (μg/g)	1252.77 ± 264.20a	1276.74 ± 142.40a	1269.89 ± 158.37a	1240.92 ± 198.85a	1476.87 ± 163.84b	***
Fru-Gln (μg/g)	919.08 ± 690.28a	346.41 ± 556.75bde	507.08 ± 421.19ce	462.28 ± 416.90cd	201.50 ± 227.37b	***
Fru-Glu (μg/g)	495.27 ± 285.35a	294.05 ± 198.46b	404.48 ± 211.81c	319.05 ± 172.19bc	267.96 ± 166.54b	***
Fru-Ile (μg/g)	23.07 ± 4.80a	20.36 ± 1.91b	24.73 ± 1.95c	22.62 ± 2.93a	27.93 ± 3.41d	***
Fru-Leu (μg/g)	50.11 ± 9.03a	41.34 ± 4.85b	46.41 ± 5.58c	40.58 ± 5.31b	46.87 ± 7.03c	***
Fru-Tyr (μg/g)	74.49 ± 18.30a	51.29 ± 3.73b	54.07 ± 6.73b	52.31 ± 5.63b	53.56 ± 6.05b	***
Fru-Phe (μg/g)	704.92 ± 176.22a	385.97 ± 116.30b	491.10 ± 133.00c	362.38 ± 83.53b	368.84 ± 88.95b	***
Fru-Trp (μg/g)	364.69 ± 173.50a	180.15 ± 109.52b	290.36 ± 120.87c	199.29 ± 99.63b	175.01 ± 95.30b	***
Neo-phytene (mg/g)	0.84 ± 0.18a	1.09 ± 0.17b	1.13 ± 0.09b	0.88 ± 0.10a	0.85 ± 0.09a	***

p-value of one-way ANOVA: ns: p-value > 0.05 (not significant), 0.05 > p-value > 0.01 (significant), 0.01 > p-value > 0.001 (highly significant) ***: p-value ≤ 0.001 (extremely significant) (Cui et al., 2023a).

Based on routine chemical components such as total alkaloids, reducing sugar, total sugar, and total nitrogen as examples, tobacco leaves from Brazil exhibited the highest total alkaloid content, with an average of 3.98%, whereas Chinese tobacco leaves showed the lowest average content of 2.50%. The reducing sugar content was the highest in the Chinese samples (20.96%) and the lowest in the United States samples (11.69%). Similarly, the total sugar content was the highest in China (23.45%) and lowest in the United States (12.33%). The highest total nitrogen content was observed in Brazil (2.65%) and the lowest in Zimbabwe (2.08%). The reason may be that the different genetic backgrounds of the flue-cured tobacco cultivars grown in these countries result in distinct metabolic tendencies. Brazil typically cultivates varieties with a strong alkaloid metabolism, leading to a higher accumulation of alkaloids and nitrogenous compounds, whereas China tends to breed low-alkaloid, high-sugar, and flue-cured tobacco cultivars (Li et al., 2013; Wu et al., 2025).

Among polyphenols compounds, chlorogenic acid and rutin showed relatively high average levels across origins. Zimbabwean tobacco had the highest average chlorogenic acid content (9.81 mg/g), whereas tobacco from the United States had the lowest (5.82 mg/g). Similarly, rutin content peaked in Zimbabwe (9.38 mg/g) and was lowest in the United States (5.25 mg/g). This may be attributed to the more active phenolic metabolism in Zimbabwean tobacco, possibly related to its plateau climate characterized by strong ultraviolet radiation and moderate water stress, which favors the accumulation of polyphenolic secondary metabolites.

Among the polyacids and higher fatty acids, malic acid was the main representative substance with the highest average content. The average malic acid content was highest in Zimbabwe, at 57.55 mg/g, whereas it was lowest in the United States (39.01 mg/g). Among the amino acids, proline exhibited the highest average content and was the most abundant. Proline levels were highest in Chinese tobacco leaves (6628.86 μg/g) and lowest in Zambia (2856.99 μg/g).

Among Amadori compounds, Fru-Pro was the most abundant. Its average content was highest in China (9223.37 μg/g) and lowest in the United States (4790.33 μg/g). Amadori compounds are the early products of the Maillard reaction and contribute significantly to tobacco quality. The high Fru-Pro levels in the Chinese samples may be associated with their elevated sugar and proline contents, reflecting favorable precursor conditions and curing processes for Fru-Pro formation.

Regarding neo-phytene, the highest content was detected in Brazil (1.13 mg/g), while the lowest was found in China (0.84 mg/g). Neo-phytene is a typical terpene compound; its higher content in Brazil may be due to its tropical climate with strong sunlight and high temperatures, which are conducive to terpene synthesis and accumulation.

Notably, although some indicators show significant differences overall, the values in certain regions are extremely close, and multiple comparison results reveal no significant differences. In terms of potassium content, the United States (2.38 ± 0.19) and Zimbabwe (2.38 ± 0.20) samples differ very little, both labeled “b,” indicating no statistical difference in potassium content between the two countries. Regarding pH, the United States (5.03 ± 0.12), Brazil (5.02 ± 0.09), and Zambia (5.04 ± 0.09) samples show minimal differences, with letter labels bd, b, and bc, respectively; the pH values of the United States samples are therefore not significantly different from those of Brazil or Zambia. For phosphate content, the United States (5.38 ± 0.40) and Zimbabwe (5.38 ± 0.40) samples have identical values, labeled be and de, respectively, indicating no significant difference. These results suggest that even for indicators exhibiting significant overall differences, regions that are geographically close or have similar ecological conditions may still display convergence in chemical composition.

Furthermore, a one-way ANOVA was conducted on tobacco leaves from nine major Chinese production regions (Yunnan, Sichuan, Guizhou, Chongqing, Henan, Hunan, Fujian, Shandong, and Heilongjiang). The results are provided in the Supplementary Table S1. The results showed that tobacco leaves from different regions in China exhibited highly significant differences in chemical composition (p-value ≤ 0.001), which may be attributed to the unique geographical locations and climatic conditions of each production area.

**FIGURE 3:**
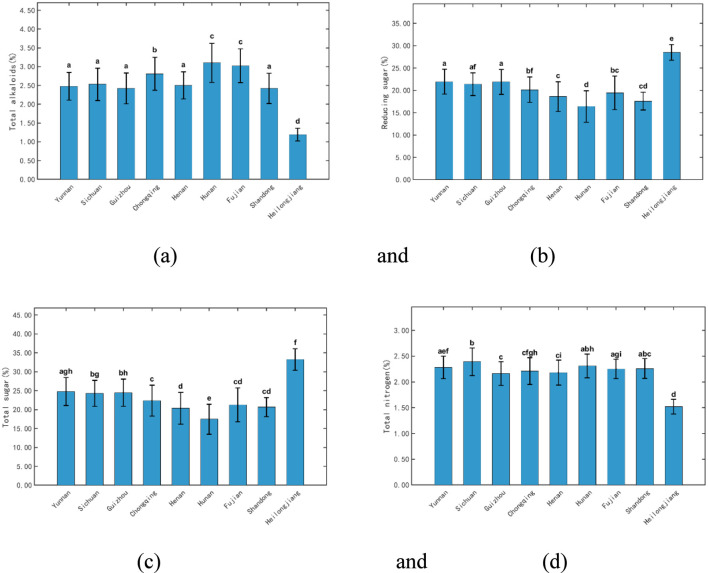
Bar plots of the mean contents ± standard deviations of **(a)** total alkaloids, **(b)** reducing sugar, **(c)** total sugar, and **(d)** total nitrogen in tobacco leaves from different regions. For the same indicator, groups sharing the same letter are not significantly different (p-value > 0.05), whereas groups with different letters are significantly different (p-value < 0.05).

To facilitate the comparison of the chemical composition differences among tobacco leaves from different regions (Yunnan, Sichuan, Guizhou, Chongqing, Henan, Hunan, Fujian, Shandong, and Heilongjiang) and to provide a visual basis for interpreting regional variations, taking routine chemical components such as total alkaloids, reducing sugar, total sugar, and total nitrogen as examples, Figures 3a–d show bar charts of the mean contents ± standard deviations for these four components. Hunan had the highest total alkaloid content (3.10%), whereas Heilongjiang had the lowest (1.19%). Heilongjiang had the highest reducing sugar and total sugar content (28.50% and 33.23%, respectively), whereas Hunan had the lowest (16.39% and 17.45%, respectively). Sichuan exhibited the highest total nitrogen content (2.39%), whereas Heilongjiang had the lowest (1.52%). These differences likely correlate with the ecological climate, soil conditions, and cultivation strategies. The warm and humid climate in Hunan favors alkaloid accumulation, whereas Heilongjiang’s cooler climate and longer growth period promote sugar preservation and accumulation while inhibiting alkaloid synthesis. Sufficient soil nitrogen supply or higher nitrogen fertilization rates in Sichuan likely promoted elevated total nitrogen content.

Tobacco leaves from all 13 regions—including Yunnan, Sichuan, Guizhou, Chongqing, Henan, Hunan, Fujian, Shandong, and Heilongjiang in China, as well as the United States, Brazil, Zimbabwe, and Zambia—were further subjected to one-way ANOVA. The results are shown in the Supplementary Table S2. The results showed that the tobacco leaves from the 13 regions exhibited highly significant differences (p-value ≤ 0.001) in terms of all 70 chemical components. These findings provide a solid foundation for subsequent studies on origin discrimination and classification based on these chemical components.

#### 3.1.2 HCA

To investigate the similarity characteristics among samples from different origins, HCA was performed based on the mean values of 70 chemical variables. The Pearson correlation coefficient was used as the distance metric to reveal potential relationships between the chemical composition and geographic origin. HCA is an unsupervised pattern recognition method that groups samples into clusters based on their similarity, thereby intuitively reflecting the intrinsic connections among samples from different regions and providing a theoretical basis for geographic tracing and origin identification.

**FIGURE 4:**
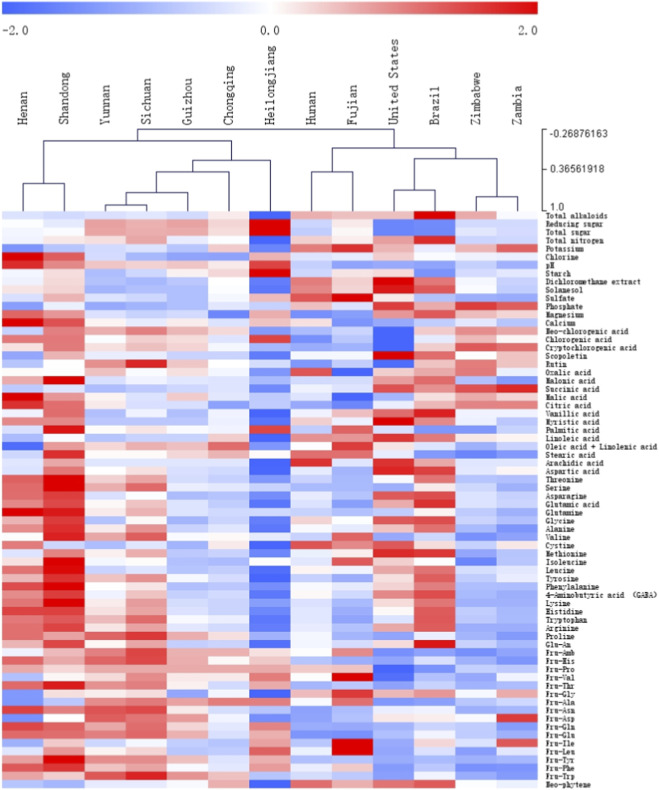
Hierarchical clustering results of tobacco leaf samples from different regions based on 70 chemical components.

The clustering dendrogram based on the chemical composition data (Figure 4) revealed clear hierarchical relationships among samples from different origins. Initially, samples from Yunnan and Sichuan clustered together as a subclass, while those from Guizhou formed another subclass; subsequently, Yunnan, Sichuan, and Guizhou merged, indicating high chemical similarity among these provinces. Chongqing clustered separately from the three aforementioned provinces; however, at a higher hierarchical level, these four provinces clustered together, reflecting a close association among samples from Southwest China. Heilongjiang, as a relatively independent subclass, further clustered with the four southwestern provinces, suggesting a cross-regional similarity. Samples from Henan and Shandong formed a cluster that merged with the aforementioned five provinces (Yunnan, Sichuan, Guizhou, Chongqing, and Heilongjiang) to create a larger cluster representing domestic Chinese samples.

Among the international samples, the United States and Brazil clustered together, while Zimbabwe and Zambia formed another subclass. These two groups subsequently merged, forming a cluster predominantly comprising international samples. Additionally, samples from Hunan and Fujian formed a distinct cluster, which was subsequently integrated into an international cluster (the United States, Brazil, Zimbabwe, and Zambia), forming another major subgroup. The overall clustering structure revealed a certain degree of consistency between the geographical origin of the samples and their chemical composition. The tobacco samples from China and other countries were grouped into major clusters. Within the Chinese samples, significant clustering patterns were observed among geographically adjacent regions. This observation is consistent with previous multi-element analyses of tobacco origin, in which samples from Henan and Shandong formed a single cluster (Cui L.-L. et al., 2023). These clustering results indicate that tobacco leaves from different regions possess distinct geographical and chemical characteristics. To some extent, the clustering structure reflects the influence of the geographical distribution on the chemical profiles of the samples and provides a scientific basis for subsequent research on origin identification and quality traceability.

### 3.2 Discrimination of tobacco geographic origins using machine learning models

Based on the results from Section 3.1, where one-way ANOVA showed highly significant differences (p < 0.001) in 70 chemical components among tobacco leaves from 13 regions, HCA revealed clear regional chemical characteristics. Tobacco leaf samples from these 13 regions were used to build origin discrimination models using SVM, BPNN, and RF algorithms.

PSO was employed to identify the optimal parameter combinations for the three machine learning models. Table 6 presents the average accuracy of the five-fold cross-validation under the optimal parameters obtained using PSO. For the SVM model, the hybrid kernel function demonstrates the best performance, achieving an accuracy of 96.51%. This may be attributed to the ability of the hybrid kernel to integrate the advantages of multiple kernel functions using a weighted combination, thereby leveraging their respective strengths and enhancing the classification capability of the model. Therefore, the hybrid kernel was selected as the final kernel function for the SVM.

**TABLE 6:** Average accuracy of five-fold cross-validation under the optimal parameters for each model.

Model	Optimal parameter	The average accuracy of five-fold cross-validation/%
SVM- linear kernel	—	93.96
SVM- polynomial kernel	*c*_1_ = 0.0125, *c*_2_ = 2.6077, *c*_3_ = 4.0000	96.00
SVM- Gaussian kernel	$\sigma^{2}$ = 20.7940	93.96
SVM-sigmoid kernel	*a* = 0.0100, *c* = −0.8030	84.72
SVM- hybrid kernel	*c*_1_ = 0.0125, *c*_2_ = 3.0000, *c*_3_ = 4.0000	96.51
	$\sigma^{2}$ = 10.0000	
	*m* = 0.0000, *n* = 0.4215, *q* = 0.5785	
BPNN	Number of hidden layers = 3, number of neurons in each layer = 31, learning rate = 0.001	95.85
RF	Number of trees = 151, minimum number of samples required for each leaf node = 2	91.63

For the BPNN model, the highest average accuracy of the five-fold cross-validation was achieved when the network had three hidden layers, each containing 31 neurons, with a learning rate of 0.001. This result can be explained by the fact that, for datasets with limited sample sizes, a deeper network with more neurons may lead to overfitting owing to excessive model complexity. The chosen configuration of three hidden layers, 31 neurons in each layer, and the learning rate of 0.001 likely strikes a balance between model complexity and generalization ability, effectively avoiding both overfitting and underfitting.

For the RF model, the optimal parameters were found to be 151 trees and a minimum of two samples for each leaf node, yielding the highest average accuracy of the five-fold cross-validation. This setting likely balances model complexity and computational efficiency, helping to prevent overfitting while enhancing model stability and generalization performance.

After optimizing each model and determining the optimal parameters, the models were trained using a training set and validated using a test set. The overall accuracies of both the training and test sets, as well as the macro-average recall, macro-average precision, and macro-average F1 score for each origin discrimination model, are summarized in Table 7. The SVM model with the hybrid kernel achieved the highest test set overall accuracy of 97.96% with macro-average recall, macro-average precision, and macro-average F1 scores of 0.9836, 0.9806, and 0.9821, respectively. Compared with the BPNN model, the SVM-hybrid kernel model improved the macro-average recall by 4.04 percentage points, macro-average precision by 0.70 percentage points, and macro-average F1 score by 2.39 percentage points. Compared with the RF model, the improvements were even more pronounced, with increases of 6.66, 3.58, and 5.14 percentage points in the macro-average recall, macro-average precision, and macro-average F1 score, respectively.

**TABLE 7:** Overall accuracy on training and test sets, macro-average recall, macro-average precision, and macro-average F1 score of origin discrimination models.

Model	Overall accuracy of the training set/%	Overall accuracy of the test set/%	Macro-average recall	Macro-average precision	Macro-average F1 score
SVM- hybrid kernel	99.85	97.96	0.9836	0.9806	0.9821
BPNN	98.98	96.79	0.9432	0.9736	0.9582
RF	99.85	93.29	0.917	0.9448	0.9307

This study found that the choice of kernel function in the SVM significantly affected the discrimination performance of tobacco origin models, with the SVM-hybrid kernel model achieving the highest accuracy. One possible reason is that the hybrid kernel function combines the characteristics of multiple kernel functions and can capture different levels and types of features in the data. For example, the linear kernel function is suitable for processing simple linear data; the polynomial kernel function is suitable for handling situations with interactions or nonlinear features; the Gaussian kernel function is capable of processing local information; and the sigmoid kernel function is similar to the activation function in a neural network, which is suitable for processing continuous nonlinear patterns. The hybrid kernel function can be weighted among these kernels, making the model more flexible and improving its ability to classify and generalize complex data. This is consistent with the results of a previous study (Zhang, 2001). It was also observed that the SVM model with the hybrid kernel outperformed both the BPNN and RF models. This may be attributed to the higher sensitivity of the neural network to parameter settings and sample size, whereas the RF model may be prone to overfitting when handling high-dimensional continuous variables.

**FIGURE 5:**
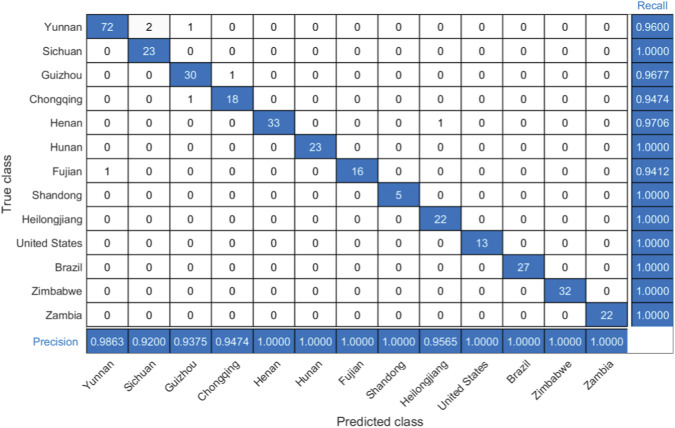
Confusion matrix of the SVM-hybrid kernel model on the test set.

Figure 5 shows the confusion matrix of the SVM-hybrid kernel model for the test set. The recall for Yunnan origin was 0.9600, with two Yunnan samples misclassified as Sichuan and one as Guizhou. The recall for Guizhou was 0.9677, because one sample was misclassified as Chongqing. Chongqing has a recall of 0.9474, with one sample misclassified as Guizhou. Henan achieved a recall of 0.9706 with one sample misclassified as Heilongjiang. Fujian’s recall was 0.9412, with one sample misclassified as Yunnan. The recall for all other origins was 1.0000, indicating perfect classification accuracy. The precision for each origin was as follows: Yunnan (0.9863), Sichuan (0.9200), Guizhou (0.9375), Chongqing (0.9474), Henan (1.0000), Hunan (1.0000), Fujian (1.0000), Shandong (1.0000), Heilongjiang (0.9565), United States (1.0000), Brazil (1.0000), Zimbabwe (1.0000), and Zambia (1.0000).

### 3.3 Chemical component importance analysis

**FIGURE 6:**
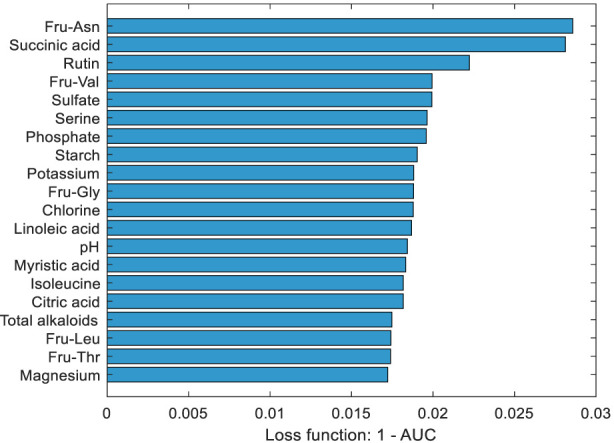
Ranking of the top 20 important chemical components based on PFI.

The PFI algorithm was applied to interpret the SVM-hybrid kernel model (optimal model) and identify the key chemical components influencing origin classification. The loss function used for PFI was defined as 1−AUC. Figure 6 shows the rankings of the 20 most important chemical components. The results indicate that Fru-Asn, succinic acid, rutin, Fru-Val, sulfate, serine, phosphate, starch, potassium, Fru-Gly, chlorine, linoleic acid, pH, myristic acid, isoleucine, citric acid, total alkaloids, Fru-Leu, Fru-Thr, and magnesium are critical chemical markers affecting the origin discrimination model. Combined with the one-way ANOVA results (Supplementary Table S2), the concentration ranges of these significant compounds across different origins were determined. Taking the top three components—Fru-Asn, succinic acid, and rutin—as examples, Fru-Asn exhibited the highest concentration in Henan (mean: 4368.55 μg/g) and the lowest in Hunan (mean: 2666.50 μg/g). Succinic acid had the highest concentration in Zambia (0.41 mg/g) and the lowest in Heilongjiang (0.22 mg/g). Rutin had the highest level in Sichuan (10.40 mg/g) and the lowest in the United States (5.25 mg/g). These variations may result from a combination of factors, including genetic background, climate, soil characteristics, cultivar differences, and postharvest processing practices. These findings are of great significance for geographical traceability, chemical characterization, and quality control of tobacco.

## 4 Conclusion

This study employed NIR spectroscopy combined with rapid chemical composition analysis technology to obtain 70 chemical components in tobacco samples from nine major tobacco-producing regions in China (Yunnan, Sichuan, Guizhou, Chongqing, Henan, Hunan, Fujian, Shandong, and Heilongjiang), as well as from four other major tobacco-producing countries (the United States, Brazil, Zimbabwe, and Zambia). These components encompass both major and trace substances in tobacco leaves and represent a crucial foundation for tobacco quality. One-way ANOVA and HCA were performed to investigate regional differences in chemical composition. Thirteen tobacco origin discrimination models were constructed using the SVM, BPNN, and RF algorithms. The best-performing model was further interpreted using the PFI method to identify key chemical markers relevant to origin classification. Except for chlorine, extremely significant differences (p-value ≤ 0.001) were found in the chemical composition of tobacco leaves from the five countries. Similarly, all Chinese tobacco regions exhibited highly significant differences. Moreover, tobacco leaves from all 13 regions exhibited highly significant differences (p-value ≤ 0.001) across all 70 chemical components. The HCA results further demonstrated distinct geographical patterns in tobacco chemical profiles. Among the models, the SVM-hybrid kernel achieved the highest performance, with a test-set accuracy of 97.96% and macro-average recall, macro-average precision, and macro-average F1 scores of 0.9836, 0.9806, and 0.9821, respectively. PFI analysis revealed that Fru-Asn, succinic acid, rutin, Fru-Val, sulfate, serine, phosphate, starch, potassium, Fru-Gly, chlorine, linoleic acid, pH, myristic acid, isoleucine, citric acid, total alkaloids, Fru-Leu, Fru-Thr, and magnesium were critical chemical markers affecting the origin discrimination model. This study combined chemometric methods, NIR and rapid chemical composition analysis technology, and interpretable machine learning to achieve rapid, stable, and accurate discrimination of tobacco origins. It also clarified the chemical characteristics of tobacco from different regions, providing new insights into the geographical traceability and chemical profiling of tobacco.

## Data Availability

The datasets presented in this article are not readily available because Author do not have permission to share. Requests to access the datasets should be directed to Ranran Kou, 3288727224@qq.com.
