# Supplementary material for: Rapid discrimination of geographical origin and analysis of chemical characterization of tobacco leaves from multiple countries
Source: Front Chem. 2026 Mar 20;14:1721371. doi: 10.3389/fchem.2026.1721371 (PMC13046703; doi:10.3389/fchem.2026.1721371)
Supplement: Supplementary file 1 [file DataSheet1.docx]

**Rapid discrimination of geographical origin and analysis of chemical characterization of tobacco leaves from multiple countries**

Ranran Kou ^1+^, Cong Wang ^1+^, Ran Wan ^1^, Mingliang Su ^2^, Heng Xu ^3^, Yufeng Fu ^3^, Yun Lin ^4^, Le Zhao ^1^, Junwei Guo ^1^, Hongbo Wang ^1^, Zechun Liu ^2*^, Song Yang ^1*^, Cong Nie ^1*^

^1^ Key Laboratory of Tobacco Chemistry, Zhengzhou Tobacco Research Institute of China National Tobacco Corporation (CNTC), Zhengzhou 450001, China

^2^ Technology Center, China Tobacco Fujian Industrial Co., Ltd., Xiamen 361021, China

^3^ Technology Center, China Tobacco Henan Industrial Co., Ltd., Zhengzhou 450000, China

^4^ Technology Center, China Tobacco Guangdong Industrial Co., Ltd., Guangzhou 510385, China

^+^ Ranran Kou and Cong Wang contributed equally to this work.

*** Correspondence:**Corresponding Author:

Zechun Liu

lzc10497@fjtic.cn

Song Yang

ztriyangs@163.com

Cong Nie

niec@ztri.com.cn

Table S1 Mean contents ± standard deviations of 70 chemical components in tobacco leaf samples from Chinese tobacco regions

| Chemical component | Yunnan | Sichuan | Guizhou | Chongqing | Henan | Hunan | Fujian | Shandong | Heilongjiang | Significance level |
| --- | --- | --- | --- | --- | --- | --- | --- | --- | --- | --- |
|  | n=360 | n=103 | n=157 | n=92 | n=206 | n=103 | n=124 | n=24 | n=100 |  |
| Total alkaloids(%) | 2.48 ± 0.37 | 2.53 ± 0.43 | 2.42 ± 0.41 | 2.81 ± 0.44 | 2.50 ± 0.36 | 3.10 ± 0.52 | 3.02 ± 0.45 | 2.42 ± 0.40 | 1.19 ± 0.17 | *** |
| Reducing sugar(%) | 21.95 ± 2.77 | 21.36 ± 2.55 | 21.90 ± 2.79 | 20.12 ± 2.86 | 18.61 ± 3.34 | 16.39 ± 3.54 | 19.42 ± 3.75 | 17.58 ± 2.00 | 28.50 ± 1.77 | *** |
| Total sugar(%) | 24.76 ± 3.69 | 24.31 ± 3.42 | 24.47 ± 3.60 | 22.37 ± 4.08 | 20.34 ± 4.18 | 17.45 ± 3.94 | 21.27 ± 4.48 | 20.66 ± 2.53 | 33.23 ± 2.85 | *** |
| Total nitrogen(%) | 2.28 ± 0.22 | 2.39 ± 0.27 | 2.16 ± 0.23 | 2.21 ± 0.26 | 2.18 ± 0.24 | 2.31 ± 0.23 | 2.25 ± 0.19 | 2.26 ± 0.19 | 1.52 ± 0.14 | *** |
| Potassium(%) | 1.91 ± 0.27 | 1.96 ± 0.24 | 1.78 ± 0.19 | 2.30 ± 0.16 | 1.49 ± 0.23 | 2.58 ± 0.23 | 2.87 ± 0.34 | 1.78 ± 0.24 | 1.40 ± 0.24 | *** |
| Chlorine(%) | 0.37 ± 0.13 | 0.25 ± 0.09 | 0.19 ± 0.06 | 0.18 ± 0.10 | 1.09 ± 0.58 | 0.40 ± 0.12 | 0.42 ± 0.12 | 0.80 ± 0.16 | 0.63 ± 0.17 | *** |
| pH | 5.19 ± 0.14 | 5.18 ± 0.15 | 5.19 ± 0.14 | 5.16 ± 0.16 | 5.32 ± 0.14 | 5.08 ± 0.10 | 5.02 ± 0.11 | 5.24 ± 0.17 | 5.30 ± 0.13 | *** |
| Starch(%) | 3.67 ± 0.66 | 3.99 ± 0.66 | 4.56 ± 0.66 | 4.74 ± 0.72 | 4.35 ± 0.94 | 3.99 ± 0.90 | 4.65 ± 1.07 | 4.70 ± 0.60 | 7.13 ± 1.07 | *** |
| Dichloromethane extract(%) | 3.98 ± 0.55 | 3.87 ± 0.38 | 4.03 ± 0.47 | 4.59 ± 0.58 | 4.46 ± 0.84 | 5.40 ± 0.76 | 4.88 ± 0.81 | 4.82 ± 0.42 | 3.27 ± 0.28 | *** |
| Solanesol(mg/g) | 7.16 ± 2.13 | 7.58 ± 2.17 | 7.54 ± 2.38 | 10.01 ± 3.16 | 10.71 ± 4.26 | 13.01 ± 3.57 | 11.12 ± 3.75 | 11.10 ± 2.25 | 4.69 ± 1.60 | *** |
| Sulfate(mg/g) | 7.44 ± 3.96 | 6.89 ± 0.81 | 5.80 ± 1.18 | 8.30 ± 1.35 | 7.31 ± 1.76 | 13.30 ± 2.63 | 16.90 ± 2.96 | 10.69 ± 1.27 | 10.50 ± 2.16 | *** |
| Phosphate(mg/g) | 3.98 ± 0.45 | 4.05 ± 0.42 | 4.13 ± 0.36 | 4.41 ± 0.36 | 3.90 ± 0.37 | 4.82 ± 0.37 | 4.37 ± 0.36 | 4.46 ± 0.44 | 4.25 ± 0.39 | *** |
| Magnesium(%) | 0.44 ± 0.08 | 0.42 ± 0.05 | 0.43 ± 0.05 | 0.32 ± 0.07 | 0.60 ± 0.17 | 0.33 ± 0.04 | 0.35 ± 0.05 | 0.56 ± 0.08 | 0.54 ± 0.06 | *** |
| Calcium(%) | 2.09 ± 0.26 | 1.92 ± 0.21 | 2.12 ± 0.24 | 1.86 ± 0.33 | 3.32 ± 0.48 | 2.17 ± 0.18 | 1.30 ± 0.32 | 2.79 ± 0.28 | 2.29 ± 0.24 | *** |
| Neo-chlorogenic acid(mg/g) | 1.40 ± 0.24 | 1.47 ± 0.21 | 1.39 ± 0.19 | 1.34 ± 0.20 | 1.18 ± 0.31 | 1.13 ± 0.22 | 1.19 ± 0.25 | 1.47 ± 0.28 | 1.05 ± 0.19 | *** |
| Chlorogenic acid(mg/g) | 8.93 ± 1.42 | 9.55 ± 1.07 | 9.39 ± 1.42 | 8.50 ± 1.66 | 10.56 ± 1.40 | 6.83 ± 1.33 | 7.90 ± 1.80 | 10.56 ± 1.35 | 11.23 ± 1.30 | *** |
| Cryptochlorogenic acid(mg/g) | 2.00 ± 0.31 | 2.07 ± 0.29 | 2.03 ± 0.26 | 1.91 ± 0.24 | 2.00 ± 0.44 | 1.65 ± 0.25 | 1.67 ± 0.32 | 2.22 ± 0.35 | 1.69 ± 0.28 | *** |
| Scopoletin(mg/g) | 0.26 ± 0.06 | 0.26 ± 0.06 | 0.23 ± 0.06 | 0.23 ± 0.08 | 0.18 ± 0.06 | 0.27 ± 0.07 | 0.27 ± 0.07 | 0.33 ± 0.09 | 0.13 ± 0.04 | *** |
| Rutin(mg/g) | 9.17 ± 2.06 | 10.40 ± 2.03 | 8.56 ± 1.80 | 7.94 ± 2.04 | 6.91 ± 1.75 | 6.05 ± 1.48 | 7.07 ± 1.55 | 7.93 ± 2.18 | 7.35 ± 1.48 | *** |
| Oxalic acid(mg/g) | 12.37 ± 1.34 | 11.50 ± 1.28 | 11.98 ± 1.62 | 11.40 ± 1.81 | 11.63 ± 2.13 | 13.80 ± 2.20 | 7.40 ± 1.80 | 11.60 ± 1.11 | 9.35 ± 1.08 | *** |
| Malonic acid(mg/g) | 1.38 ± 0.45 | 1.46 ± 0.43 | 1.41 ± 0.49 | 1.40 ± 0.55 | 1.72 ± 0.40 | 1.36 ± 0.48 | 1.37 ± 0.46 | 2.30 ± 0.49 | 1.22 ± 0.34 | *** |
| Succinic acid(mg/g) | 0.26 ± 0.03 | 0.27 ± 0.04 | 0.28 ± 0.03 | 0.29 ± 0.05 | 0.27 ± 0.03 | 0.30 ± 0.04 | 0.29 ± 0.06 | 0.29 ± 0.03 | 0.22 ± 0.03 | *** |
| Malic acid(mg/g) | 51.15 ± 11.30 | 45.84 ± 7.40 | 54.69 ± 7.46 | 46.72 ± 7.59 | 74.51 ± 14.23 | 46.15 ± 5.66 | 25.17 ± 8.87 | 59.43 ± 7.23 | 42.31 ± 7.52 | *** |
| Citric acid(mg/g) | 6.82 ± 1.41 | 5.95 ± 0.79 | 5.96 ± 0.94 | 4.67 ± 1.35 | 9.16 ± 2.22 | 4.85 ± 0.94 | 4.12 ± 1.29 | 8.36 ± 1.23 | 5.55 ± 0.94 | *** |
| Vanillic acid(mg/g) | 0.12 ± 0.01 | 0.13 ± 0.01 | 0.12 ± 0.01 | 0.13 ± 0.01 | 0.13 ± 0.01 | 0.13 ± 0.01 | 0.14 ± 0.01 | 0.15 ± 0.01 | 0.10 ± 0.01 | *** |
| Myristic acid(mg/g) | 0.14 ± 0.01 | 0.14 ± 0.01 | 0.14 ± 0.02 | 0.15 ± 0.01 | 0.17 ± 0.02 | 0.16 ± 0.02 | 0.15 ± 0.02 | 0.17 ± 0.02 | 0.12 ± 0.01 | *** |
| Palmitic acid(mg/g) | 2.83 ± 0.13 | 2.85 ± 0.10 | 2.82 ± 0.10 | 2.81 ± 0.10 | 2.78 ± 0.17 | 2.75 ± 0.11 | 2.97 ± 0.15 | 3.05 ± 0.10 | 3.00 ± 0.08 | *** |
| Linoleic acid(mg/g) | 1.66 ± 0.14 | 1.70 ± 0.11 | 1.70 ± 0.12 | 1.92 ± 0.17 | 1.63 ± 0.21 | 2.03 ± 0.18 | 2.02 ± 0.22 | 1.82 ± 0.15 | 1.32 ± 0.09 | *** |
| Oleic acid + Linolenic acid(mg/g) | 3.68 ± 0.31 | 3.76 ± 0.29 | 3.73 ± 0.29 | 3.91 ± 0.36 | 3.07 ± 0.44 | 3.59 ± 0.28 | 4.02 ± 0.32 | 3.81 ± 0.27 | 3.22 ± 0.23 | *** |
| Stearic acid(mg/g) | 0.56 ± 0.03 | 0.56 ± 0.04 | 0.57 ± 0.02 | 0.58 ± 0.02 | 0.54 ± 0.06 | 0.60 ± 0.03 | 0.60 ± 0.03 | 0.62 ± 0.03 | 0.56 ± 0.02 | *** |
| Arachidic acid(mg/g) | 0.13 ± 0.01 | 0.13 ± 0.01 | 0.13 ± 0.01 | 0.13 ± 0.01 | 0.13 ± 0.01 | 0.15 ± 0.01 | 0.13 ± 0.01 | 0.14 ± 0.01 | 0.11 ± 0.01 | *** |
| Aspartic acid(μg/g) | 317.14 ± 99.44 | 339.08 ± 78.30 | 279.59 ± 69.77 | 271.59 ± 96.18 | 283.06 ± 92.44 | 293.50 ± 77.87 | 226.36 ± 80.87 | 417.63 ± 70.29 | 128.62 ± 49.10 | *** |
| Threonine(μg/g) | 50.25 ± 25.06 | 57.25 ± 22.10 | 34.12 ± 20.01 | 30.98 ± 23.18 | 68.41 ± 35.12 | 31.51 ± 15.96 | 24.73 ± 13.85 | 84.23 ± 24.86 | 15.56 ± 10.21 | *** |
| Serine(μg/g) | 168.31 ± 92.99 | 197.27 ± 81.94 | 124.64 ± 72.91 | 121.59 ± 84.33 | 196.61 ± 99.94 | 89.98 ± 53.25 | 89.45 ± 55.66 | 247.57 ± 109.69 | 106.52 ± 58.71 | *** |
| Asparagine(μg/g) | 1075.29 ± 615.74 | 1299.39 ± 591.66 | 765.60 ± 564.30 | 835.43 ± 691.96 | 2041.56 ± 1118.44 | 1161.45 ± 566.62 | 831.50 ± 481.66 | 2286.47 ± 618.40 | 368.45 ± 345.62 | *** |
| Glutamic acid(μg/g) | 157.70 ± 80.91 | 185.08 ± 74.73 | 131.56 ± 67.29 | 110.54 ± 75.89 | 215.68 ± 107.38 | 130.88 ± 57.93 | 72.28 ± 50.56 | 249.07 ± 80.75 | 35.90 ± 28.76 | *** |
| Glutamine(μg/g) | 414.35 ± 297.60 | 525.32 ± 273.52 | 292.56 ± 248.27 | 256.77 ± 270.26 | 925.77 ± 496.99 | 236.03 ± 183.69 | 176.41 ± 163.39 | 844.62 ± 329.46 | 256.67 ± 188.22 | *** |
| Glycine(μg/g) | 23.22 ± 6.72 | 29.07 ± 7.99 | 21.91 ± 6.47 | 24.90 ± 9.10 | 32.79 ± 12.26 | 31.04 ± 8.29 | 28.51 ± 7.59 | 39.70 ± 9.40 | 16.95 ± 3.83 | *** |
| Alanine(μg/g) | 302.12 ± 106.71 | 347.33 ± 100.26 | 253.58 ± 97.85 | 270.64 ± 114.78 | 350.77 ± 133.08 | 286.03 ± 78.06 | 274.07 ± 69.59 | 402.43 ± 114.15 | 173.94 ± 56.43 | *** |
| Valine(μg/g) | 312.38 ± 61.61 | 338.31 ± 51.57 | 279.92 ± 49.68 | 284.55 ± 62.33 | 283.31 ± 53.46 | 256.88 ± 46.05 | 336.29 ± 52.25 | 362.10 ± 65.89 | 270.82 ± 41.45 | *** |
| Cystine(μg/g) | 84.15 ± 10.92 | 86.96 ± 7.24 | 83.54 ± 6.21 | 89.53 ± 8.61 | 94.08 ± 9.25 | 95.77 ± 6.99 | 93.49 ± 8.82 | 85.91 ± 7.36 | 77.87 ± 5.45 | *** |
| Methionine(μg/g) | 13.19 ± 2.87 | 14.38 ± 3.25 | 11.29 ± 3.01 | 11.56 ± 2.95 | 12.15 ± 2.88 | 12.48 ± 2.95 | 13.23 ± 2.52 | 15.18 ± 3.17 | 9.28 ± 1.49 | *** |
| Isoleucine(μg/g) | 9.66 ± 1.93 | 9.93 ± 1.59 | 8.16 ± 1.34 | 8.02 ± 1.99 | 10.36 ± 2.43 | 9.24 ± 2.01 | 12.61 ± 2.10 | 14.86 ± 2.79 | 9.90 ± 1.89 | *** |
| Leucine(μg/g) | 16.13 ± 3.30 | 16.90 ± 2.85 | 15.47 ± 3.06 | 15.91 ± 2.98 | 21.54 ± 5.22 | 18.22 ± 2.79 | 17.48 ± 2.87 | 24.47 ± 3.62 | 11.79 ± 2.75 | *** |
| Tyrosine(μg/g) | 60.92 ± 15.56 | 65.71 ± 11.75 | 53.90 ± 10.54 | 49.39 ± 12.83 | 62.00 ± 14.67 | 47.56 ± 8.99 | 41.82 ± 9.30 | 75.78 ± 10.96 | 28.87 ± 9.44 | *** |
| Phenylalanine(μg/g) | 146.33 ± 50.92 | 157.45 ± 50.91 | 103.41 ± 45.19 | 109.01 ± 57.22 | 223.34 ± 90.07 | 142.66 ± 39.53 | 132.76 ± 38.14 | 254.70 ± 49.05 | 67.74 ± 31.00 | *** |
| 4-Aminobutyric acid (GABA)(μg/g) | 100.10 ± 46.22 | 123.92 ± 44.23 | 83.54 ± 42.81 | 88.28 ± 49.97 | 140.19 ± 63.79 | 108.15 ± 41.63 | 88.15 ± 34.68 | 164.70 ± 56.58 | 39.81 ± 17.12 | *** |
| Lysine(μg/g) | 26.07 ± 13.84 | 33.16 ± 14.37 | 19.50 ± 12.08 | 18.15 ± 13.31 | 36.52 ± 19.80 | 20.09 ± 9.63 | 14.20 ± 6.01 | 47.65 ± 13.82 | 10.00 ± 3.58 | *** |
| Histidine(μg/g) | 111.52 ± 51.86 | 131.22 ± 50.33 | 86.95 ± 45.86 | 80.01 ± 50.09 | 163.89 ± 75.42 | 89.88 ± 37.21 | 59.89 ± 27.45 | 164.85 ± 46.73 | 37.81 ± 17.83 | *** |
| Tryptophan(μg/g) | 114.28 ± 58.65 | 136.31 ± 51.96 | 93.29 ± 49.26 | 88.60 ± 55.30 | 132.09 ± 64.02 | 85.32 ± 39.23 | 55.01 ± 28.48 | 141.43 ± 59.17 | 34.52 ± 13.96 | *** |
| Arginine(μg/g) | 36.22 ± 11.32 | 41.84 ± 12.16 | 31.13 ± 11.19 | 29.24 ± 12.17 | 45.93 ± 15.82 | 31.68 ± 9.31 | 25.84 ± 7.44 | 49.90 ± 11.68 | 16.52 ± 6.15 | *** |
| Proline(μg/g) | 7875.98 ± 2671.54 | 8617.34 ± 2262.90 | 6688.22 ± 2158.81 | 5937.64 ± 2080.09 | 7710.08 ± 2684.20 | 5265.17 ± 1468.14 | 3586.99 ± 1178.12 | 6913.47 ± 1908.19 | 3514.66 ± 1145.98 | *** |
| Glu-An(μg/g) | 240.65 ± 105.14 | 291.44 ± 112.78 | 182.93 ± 101.97 | 189.30 ± 114.14 | 282.26 ± 121.68 | 224.02 ± 97.41 | 197.07 ± 76.16 | 357.25 ± 76.64 | 77.81 ± 50.90 | *** |
| Fru-Amb(μg/g) | 2323.13 ± 423.66 | 2458.82 ± 283.73 | 2207.41 ± 264.83 | 2210.11 ± 330.68 | 1991.31 ± 328.34 | 2043.46 ± 299.83 | 2297.63 ± 303.57 | 2189.92 ± 319.76 | 2103.88 ± 233.47 | *** |
| Fru-His(μg/g) | 80.76 ± 36.07 | 85.78 ± 31.76 | 67.92 ± 29.23 | 55.25 ± 28.98 | 80.29 ± 28.08 | 37.96 ± 21.98 | 31.52 ± 19.83 | 68.64 ± 29.57 | 63.46 ± 18.86 | *** |
| Fru-Pro(μg/g) | 9223.37 ± 1697.31 | 9223.37 ± 1336.34 | 9223.37 ± 1377.59 | 9223.37 ± 1578.30 | 9223.37 ± 2129.71 | 8874.77 ± 1733.22 | 8951.76 ± 1965.62 | 8738.70 ± 1327.05 | 9223.37 ± 1185.44 | *** |
| Fru-Val(μg/g) | 208.71 ± 28.57 | 228.46 ± 34.09 | 205.45 ± 29.52 | 204.78 ± 32.47 | 174.12 ± 36.62 | 200.30 ± 47.08 | 264.36 ± 49.59 | 201.74 ± 46.89 | 232.15 ± 36.01 | *** |
| Fru-Thr(μg/g) | 17.42 ± 4.45 | 17.89 ± 3.68 | 15.13 ± 3.26 | 13.28 ± 2.96 | 18.42 ± 4.00 | 11.82 ± 2.67 | 12.55 ± 2.74 | 20.49 ± 4.09 | 16.82 ± 2.55 | *** |
| Fru-Gly(μg/g) | 27.94 ± 3.80 | 28.36 ± 4.25 | 23.92 ± 2.96 | 27.15 ± 3.79 | 23.30 ± 3.05 | 29.22 ± 2.75 | 32.57 ± 3.45 | 28.34 ± 3.70 | 20.62 ± 1.18 | *** |
| Fru-Ala(μg/g) | 2455.04 ± 238.34 | 2495.40 ± 164.72 | 2415.42 ± 152.37 | 2491.24 ± 245.80 | 1877.69 ± 369.84 | 2209.43 ± 233.75 | 2517.16 ± 288.46 | 2044.37 ± 242.55 | 2486.68 ± 149.38 | *** |
| Fru-Asn(μg/g) | 4267.34 ± 870.88 | 4322.83 ± 847.97 | 3523.59 ± 635.72 | 3149.42 ± 845.64 | 4368.55 ± 846.53 | 2666.50 ± 550.95 | 2886.06 ± 471.51 | 4048.61 ± 642.60 | 2954.77 ± 541.95 | *** |
| Fru-Asp(μg/g) | 1433.47 ± 220.07 | 1406.79 ± 174.64 | 1364.62 ± 160.62 | 1229.84 ± 243.84 | 1045.01 ± 230.74 | 1081.31 ± 178.23 | 1136.78 ± 196.74 | 1250.51 ± 166.68 | 1038.09 ± 215.79 | *** |
| Fru-Gln(μg/g) | 1009.79 ± 698.58 | 1208.83 ± 589.65 | 868.32 ± 604.40 | 618.88 ± 567.12 | 1343.27 ± 676.37 | 292.32 ± 316.52 | 282.84 ± 319.24 | 1242.83 ± 698.63 | 1132.88 ± 496.37 | *** |
| Fru-Glu(μg/g) | 585.32 ± 297.65 | 615.00 ± 260.75 | 521.09 ± 253.71 | 362.09 ± 231.89 | 612.80 ± 225.98 | 208.45 ± 187.84 | 199.44 ± 149.23 | 608.35 ± 238.89 | 522.83 ± 167.15 | *** |
| Fru-Ile(μg/g) | 22.12 ± 3.48 | 23.44 ± 3.90 | 21.77 ± 3.28 | 21.34 ± 3.11 | 20.58 ± 3.27 | 21.47 ± 4.20 | 31.17 ± 4.64 | 25.01 ± 3.39 | 26.07 ± 4.86 | *** |
| Fru-Leu(μg/g) | 49.44 ± 5.66 | 50.92 ± 6.24 | 44.06 ± 5.23 | 44.54 ± 6.08 | 46.77 ± 7.73 | 47.62 ± 8.34 | 64.45 ± 9.34 | 54.14 ± 8.31 | 56.99 ± 7.95 | *** |
| Fru-Tyr(μg/g) | 81.70 ± 19.02 | 81.80 ± 14.32 | 75.64 ± 13.67 | 62.57 ± 10.43 | 79.33 ± 15.95 | 54.97 ± 8.39 | 55.92 ± 10.15 | 99.62 ± 18.42 | 77.26 ± 12.13 | *** |
| Fru-Phe(μg/g) | 699.99 ± 147.82 | 744.32 ± 133.42 | 606.45 ± 138.18 | 569.09 ± 144.32 | 853.17 ± 178.15 | 579.64 ± 125.14 | 690.03 ± 166.60 | 904.79 ± 200.09 | 755.76 ± 128.10 | *** |
| Fru-Trp(μg/g) | 434.30 ± 189.07 | 479.44 ± 158.28 | 386.06 ± 153.43 | 350.67 ± 163.16 | 373.11 ± 138.81 | 260.77 ± 129.88 | 236.52 ± 106.35 | 341.58 ± 158.27 | 229.38 ± 92.59 | *** |
| Neo-phytene(mg/g) | 0.84 ± 0.09 | 0.84 ± 0.09 | 0.87 ± 0.09 | 1.00 ± 0.11 | 0.74 ± 0.11 | 1.10 ± 0.10 | 1.00 ± 0.13 | 0.71 ± 0.07 | 0.44 ± 0.06 | *** |

p-value of one-way ANOVA: ns: p-value > 0.05, *:0.05 > p-value > 0.01, **: 0.01 > p-value > 0.001, ***: p-value ≤ 0.001.

Table S2 Mean contents ± standard deviations of 70 chemical components in tobacco leaf samples from 13 tobacco regions

| Chemical component | Yunnan | Sichuan | Guizhou | Chongqing | Henan | Hunan | Fujian | Shandong | Heilongjiang | United States | Brazil | Zimbabwe | Zambia | Significance level |
| --- | --- | --- | --- | --- | --- | --- | --- | --- | --- | --- | --- | --- | --- | --- |
|  | n=360 | n=103 | n=157 | n=92 | n=206 | n=103 | n=124 | n=24 | n=100 | n=64 | n=129 | n=154 | n=101 |  |
| Total alkaloids(%) | 2.48 ± 0.37 | 2.53 ± 0.43 | 2.42 ± 0.41 | 2.81 ± 0.44 | 2.50 ± 0.36 | 3.10 ± 0.52 | 3.02 ± 0.45 | 2.42 ± 0.40 | 1.19 ± 0.17 | 3.02 ± 0.23 | 3.98 ± 0.39 | 3.13 ± 0.38 | 2.68 ± 0.33 | *** |
| Reducing sugar(%) | 21.95 ± 2.77 | 21.36 ± 2.55 | 21.90 ± 2.79 | 20.12 ± 2.86 | 18.61 ± 3.34 | 16.39 ± 3.54 | 19.42 ± 3.75 | 17.58 ± 2.00 | 28.50 ± 1.77 | 11.69 ± 2.62 | 12.02 ± 1.52 | 16.63 ± 2.29 | 16.69 ± 2.40 | *** |
| Total sugar(%) | 24.76 ± 3.69 | 24.31 ± 3.42 | 24.47 ± 3.60 | 22.37 ± 4.08 | 20.34 ± 4.18 | 17.45 ± 3.94 | 21.27 ± 4.48 | 20.66 ± 2.53 | 33.23 ± 2.85 | 12.33 ± 3.21 | 12.70 ± 1.95 | 19.35 ± 3.10 | 18.47 ± 2.82 | *** |
| Total nitrogen(%) | 2.28 ± 0.22 | 2.39 ± 0.27 | 2.16 ± 0.23 | 2.21 ± 0.26 | 2.18 ± 0.24 | 2.31 ± 0.23 | 2.25 ± 0.19 | 2.26 ± 0.19 | 1.52 ± 0.14 | 2.41 ± 0.19 | 2.65 ± 0.17 | 2.08 ± 0.17 | 2.09 ± 0.19 | *** |
| Potassium(%) | 1.91 ± 0.27 | 1.96 ± 0.24 | 1.78 ± 0.19 | 2.30 ± 0.16 | 1.49 ± 0.23 | 2.58 ± 0.23 | 2.87 ± 0.34 | 1.78 ± 0.24 | 1.40 ± 0.24 | 2.38 ± 0.19 | 2.02 ± 0.16 | 2.38 ± 0.20 | 2.68 ± 0.16 | *** |
| Chlorine(%) | 0.37 ± 0.13 | 0.25 ± 0.09 | 0.19 ± 0.06 | 0.18 ± 0.10 | 1.09 ± 0.58 | 0.40 ± 0.12 | 0.42 ± 0.12 | 0.80 ± 0.16 | 0.63 ± 0.17 | 0.58 ± 0.12 | 0.47 ± 0.10 | 0.44 ± 0.18 | 0.43 ± 0.13 | *** |
| pH | 5.19 ± 0.14 | 5.18 ± 0.15 | 5.19 ± 0.14 | 5.16 ± 0.16 | 5.32 ± 0.14 | 5.08 ± 0.10 | 5.02 ± 0.11 | 5.24 ± 0.17 | 5.30 ± 0.13 | 5.03 ± 0.12 | 5.02 ± 0.09 | 5.08 ± 0.13 | 5.04 ± 0.09 | *** |
| Starch(%) | 3.67 ± 0.66 | 3.99 ± 0.66 | 4.56 ± 0.66 | 4.74 ± 0.72 | 4.35 ± 0.94 | 3.99 ± 0.90 | 4.65 ± 1.07 | 4.70 ± 0.60 | 7.13 ± 1.07 | 4.71 ± 0.87 | 3.17 ± 0.49 | 4.04 ± 0.59 | 4.20 ± 0.64 | *** |
| Dichloromethane extract(%) | 3.98 ± 0.55 | 3.87 ± 0.38 | 4.03 ± 0.47 | 4.59 ± 0.58 | 4.46 ± 0.84 | 5.40 ± 0.76 | 4.88 ± 0.81 | 4.82 ± 0.42 | 3.27 ± 0.28 | 6.28 ± 0.82 | 5.42 ± 0.42 | 4.52 ± 0.46 | 4.14 ± 0.51 | *** |
| Solanesol(mg/g) | 7.16 ± 2.13 | 7.58 ± 2.17 | 7.54 ± 2.38 | 10.01 ± 3.16 | 10.71 ± 4.26 | 13.01 ± 3.57 | 11.12 ± 3.75 | 11.10 ± 2.25 | 4.69 ± 1.60 | 15.87 ± 3.53 | 14.11 ± 2.32 | 9.95 ± 2.10 | 8.16 ± 2.35 | *** |
| Sulfate(mg/g) | 7.44 ± 3.96 | 6.89 ± 0.81 | 5.80 ± 1.18 | 8.30 ± 1.35 | 7.31 ± 1.76 | 13.30 ± 2.63 | 16.90 ± 2.96 | 10.69 ± 1.27 | 10.50 ± 2.16 | 9.30 ± 1.31 | 5.94 ± 1.08 | 5.12 ± 1.14 | 4.91 ± 1.16 | *** |
| Phosphate(mg/g) | 3.98 ± 0.45 | 4.05 ± 0.42 | 4.13 ± 0.36 | 4.41 ± 0.36 | 3.90 ± 0.37 | 4.82 ± 0.37 | 4.37 ± 0.36 | 4.46 ± 0.44 | 4.25 ± 0.39 | 5.38 ± 0.40 | 4.90 ± 0.30 | 5.38 ± 0.40 | 5.23 ± 0.35 | *** |
| Magnesium(%) | 0.44 ± 0.08 | 0.42 ± 0.05 | 0.43 ± 0.05 | 0.32 ± 0.07 | 0.60 ± 0.17 | 0.33 ± 0.04 | 0.35 ± 0.05 | 0.56 ± 0.08 | 0.54 ± 0.06 | 0.56 ± 0.06 | 0.61 ± 0.06 | 0.53 ± 0.06 | 0.51 ± 0.04 | *** |
| Calcium(%) | 2.09 ± 0.26 | 1.92 ± 0.21 | 2.12 ± 0.24 | 1.86 ± 0.33 | 3.32 ± 0.48 | 2.17 ± 0.18 | 1.30 ± 0.32 | 2.79 ± 0.28 | 2.29 ± 0.24 | 1.46 ± 0.22 | 1.70 ± 0.17 | 1.90 ± 0.22 | 1.64 ± 0.24 | *** |
| Neo-chlorogenic acid(mg/g) | 1.40 ± 0.24 | 1.47 ± 0.21 | 1.39 ± 0.19 | 1.34 ± 0.20 | 1.18 ± 0.31 | 1.13 ± 0.22 | 1.19 ± 0.25 | 1.47 ± 0.28 | 1.05 ± 0.19 | 0.89 ± 0.15 | 1.36 ± 0.16 | 1.45 ± 0.23 | 1.41 ± 0.28 | *** |
| Chlorogenic acid(mg/g) | 8.93 ± 1.42 | 9.55 ± 1.07 | 9.39 ± 1.42 | 8.50 ± 1.66 | 10.56 ± 1.40 | 6.83 ± 1.33 | 7.90 ± 1.80 | 10.56 ± 1.35 | 11.23 ± 1.30 | 5.82 ± 1.30 | 8.61 ± 1.17 | 9.81 ± 1.37 | 9.10 ± 1.62 | *** |
| Cryptochlorogenic acid(mg/g) | 2.00 ± 0.31 | 2.07 ± 0.29 | 2.03 ± 0.26 | 1.91 ± 0.24 | 2.00 ± 0.44 | 1.65 ± 0.25 | 1.67 ± 0.32 | 2.22 ± 0.35 | 1.69 ± 0.28 | 1.36 ± 0.13 | 2.04 ± 0.21 | 2.29 ± 0.31 | 2.24 ± 0.38 | *** |
| Scopoletin(mg/g) | 0.26 ± 0.06 | 0.26 ± 0.06 | 0.23 ± 0.06 | 0.23 ± 0.08 | 0.18 ± 0.06 | 0.27 ± 0.07 | 0.27 ± 0.07 | 0.33 ± 0.09 | 0.13 ± 0.04 | 0.48 ± 0.08 | 0.38 ± 0.05 | 0.28 ± 0.07 | 0.32 ± 0.06 | *** |
| Rutin(mg/g) | 9.17 ± 2.06 | 10.40 ± 2.03 | 8.56 ± 1.80 | 7.94 ± 2.04 | 6.91 ± 1.75 | 6.05 ± 1.48 | 7.07 ± 1.55 | 7.93 ± 2.18 | 7.35 ± 1.48 | 5.25 ± 1.82 | 8.60 ± 1.30 | 9.38 ± 1.75 | 8.59 ± 1.50 | *** |
| Oxalic acid(mg/g) | 12.37 ± 1.34 | 11.50 ± 1.28 | 11.98 ± 1.62 | 11.40 ± 1.81 | 11.63 ± 2.13 | 13.80 ± 2.20 | 7.40 ± 1.80 | 11.60 ± 1.11 | 9.35 ± 1.08 | 12.20 ± 1.31 | 12.67 ± 1.12 | 13.29 ± 1.24 | 11.26 ± 1.26 | *** |
| Malonic acid(mg/g) | 1.38 ± 0.45 | 1.46 ± 0.43 | 1.41 ± 0.49 | 1.40 ± 0.55 | 1.72 ± 0.40 | 1.36 ± 0.48 | 1.37 ± 0.46 | 2.30 ± 0.49 | 1.22 ± 0.34 | 1.76 ± 0.56 | 1.89 ± 0.49 | 1.25 ± 0.37 | 1.08 ± 0.35 | *** |
| Succinic acid(mg/g) | 0.26 ± 0.03 | 0.27 ± 0.04 | 0.28 ± 0.03 | 0.29 ± 0.05 | 0.27 ± 0.03 | 0.30 ± 0.04 | 0.29 ± 0.06 | 0.29 ± 0.03 | 0.22 ± 0.03 | 0.39 ± 0.04 | 0.36 ± 0.04 | 0.39 ± 0.04 | 0.41 ± 0.04 | *** |
| Malic acid(mg/g) | 51.15 ± 11.30 | 45.84 ± 7.40 | 54.69 ± 7.46 | 46.72 ± 7.59 | 74.51 ± 14.23 | 46.15 ± 5.66 | 25.17 ± 8.87 | 59.43 ± 7.23 | 42.31 ± 7.52 | 39.01 ± 5.34 | 52.87 ± 5.32 | 57.55 ± 5.64 | 53.89 ± 4.57 | *** |
| Citric acid(mg/g) | 6.82 ± 1.41 | 5.95 ± 0.79 | 5.96 ± 0.94 | 4.67 ± 1.35 | 9.16 ± 2.22 | 4.85 ± 0.94 | 4.12 ± 1.29 | 8.36 ± 1.23 | 5.55 ± 0.94 | 6.24 ± 1.01 | 7.44 ± 0.99 | 8.07 ± 1.19 | 8.09 ± 1.62 | *** |
| Vanillic acid(mg/g) | 0.12 ± 0.01 | 0.13 ± 0.01 | 0.12 ± 0.01 | 0.13 ± 0.01 | 0.13 ± 0.01 | 0.13 ± 0.01 | 0.14 ± 0.01 | 0.15 ± 0.01 | 0.10 ± 0.01 | 0.15 ± 0.01 | 0.16 ± 0.01 | 0.13 ± 0.01 | 0.13 ± 0.01 | *** |
| Myristic acid(mg/g) | 0.14 ± 0.01 | 0.14 ± 0.01 | 0.14 ± 0.02 | 0.15 ± 0.01 | 0.17 ± 0.02 | 0.16 ± 0.02 | 0.15 ± 0.02 | 0.17 ± 0.02 | 0.12 ± 0.01 | 0.19 ± 0.02 | 0.17 ± 0.01 | 0.15 ± 0.01 | 0.14 ± 0.01 | *** |
| Palmitic acid(mg/g) | 2.83 ± 0.13 | 2.85 ± 0.10 | 2.82 ± 0.10 | 2.81 ± 0.10 | 2.78 ± 0.17 | 2.75 ± 0.11 | 2.97 ± 0.15 | 3.05 ± 0.10 | 3.00 ± 0.08 | 2.78 ± 0.07 | 2.67 ± 0.09 | 2.67 ± 0.11 | 2.76 ± 0.13 | *** |
| Linoleic acid(mg/g) | 1.66 ± 0.14 | 1.70 ± 0.11 | 1.70 ± 0.12 | 1.92 ± 0.17 | 1.63 ± 0.21 | 2.03 ± 0.18 | 2.02 ± 0.22 | 1.82 ± 0.15 | 1.32 ± 0.09 | 2.20 ± 0.19 | 2.07 ± 0.10 | 1.87 ± 0.09 | 1.85 ± 0.12 | *** |
| Oleic acid + Linolenic acid(mg/g) | 3.68 ± 0.31 | 3.76 ± 0.29 | 3.73 ± 0.29 | 3.91 ± 0.36 | 3.07 ± 0.44 | 3.59 ± 0.28 | 4.02 ± 0.32 | 3.81 ± 0.27 | 3.22 ± 0.23 | 3.65 ± 0.29 | 3.50 ± 0.21 | 3.32 ± 0.24 | 3.44 ± 0.34 | *** |
| Stearic acid(mg/g) | 0.56 ± 0.03 | 0.56 ± 0.04 | 0.57 ± 0.02 | 0.58 ± 0.02 | 0.54 ± 0.06 | 0.60 ± 0.03 | 0.60 ± 0.03 | 0.62 ± 0.03 | 0.56 ± 0.02 | 0.55 ± 0.02 | 0.51 ± 0.03 | 0.50 ± 0.03 | 0.51 ± 0.04 | *** |
| Arachidic acid(mg/g) | 0.13 ± 0.01 | 0.13 ± 0.01 | 0.13 ± 0.01 | 0.13 ± 0.01 | 0.13 ± 0.01 | 0.15 ± 0.01 | 0.13 ± 0.01 | 0.14 ± 0.01 | 0.11 ± 0.01 | 0.15 ± 0.01 | 0.14 ± 0.01 | 0.13 ± 0.01 | 0.13 ± 0.01 | *** |
| Aspartic acid(μg/g) | 317.14 ± 99.44 | 339.08 ± 78.30 | 279.59 ± 69.77 | 271.59 ± 96.18 | 283.06 ± 92.44 | 293.50 ± 77.87 | 226.36 ± 80.87 | 417.63 ± 70.29 | 128.62 ± 49.10 | 484.22 ± 119.21 | 460.61 ± 69.57 | 290.94 ± 83.59 | 323.89 ± 67.00 | *** |
| Threonine(μg/g) | 50.25 ± 25.06 | 57.25 ± 22.10 | 34.12 ± 20.01 | 30.98 ± 23.18 | 68.41 ± 35.12 | 31.51 ± 15.96 | 24.73 ± 13.85 | 84.23 ± 24.86 | 15.56 ± 10.21 | 41.32 ± 27.21 | 64.90 ± 22.72 | 27.46 ± 17.39 | 26.75 ± 15.69 | *** |
| Serine(μg/g) | 168.31 ± 92.99 | 197.27 ± 81.94 | 124.64 ± 72.91 | 121.59 ± 84.33 | 196.61 ± 99.94 | 89.98 ± 53.25 | 89.45 ± 55.66 | 247.57 ± 109.69 | 106.52 ± 58.71 | 82.51 ± 87.73 | 144.52 ± 72.91 | 110.19 ± 76.17 | 84.37 ± 53.62 | *** |
| Asparagine(μg/g) | 1075.29 ± 615.74 | 1299.39 ± 591.66 | 765.60 ± 564.30 | 835.43 ± 691.96 | 2041.56 ± 1118.44 | 1161.45 ± 566.62 | 831.50 ± 481.66 | 2286.47 ± 618.40 | 368.45 ± 345.62 | 2141.25 ± 868.02 | 2204.86 ± 606.44 | 1094.49 ± 541.48 | 938.55 ± 502.07 | *** |
| Glutamic acid(μg/g) | 157.70 ± 80.91 | 185.08 ± 74.73 | 131.56 ± 67.29 | 110.54 ± 75.89 | 215.68 ± 107.38 | 130.88 ± 57.93 | 72.28 ± 50.56 | 249.07 ± 80.75 | 35.90 ± 28.76 | 196.72 ± 97.37 | 263.15 ± 74.66 | 130.45 ± 62.88 | 118.91 ± 53.07 | *** |
| Glutamine(μg/g) | 414.35 ± 297.60 | 525.32 ± 273.52 | 292.56 ± 248.27 | 256.77 ± 270.26 | 925.77 ± 496.99 | 236.03 ± 183.69 | 176.41 ± 163.39 | 844.62 ± 329.46 | 256.67 ± 188.22 | 217.61 ± 313.55 | 399.83 ± 258.08 | 212.29 ± 212.97 | 129.82 ± 144.69 | *** |
| Glycine(μg/g) | 23.22 ± 6.72 | 29.07 ± 7.99 | 21.91 ± 6.47 | 24.90 ± 9.10 | 32.79 ± 12.26 | 31.04 ± 8.29 | 28.51 ± 7.59 | 39.70 ± 9.40 | 16.95 ± 3.83 | 37.87 ± 13.20 | 38.51 ± 7.87 | 24.76 ± 8.25 | 22.99 ± 7.97 | *** |
| Alanine(μg/g) | 302.12 ± 106.71 | 347.33 ± 100.26 | 253.58 ± 97.85 | 270.64 ± 114.78 | 350.77 ± 133.08 | 286.03 ± 78.06 | 274.07 ± 69.59 | 402.43 ± 114.15 | 173.94 ± 56.43 | 322.16 ± 124.02 | 364.98 ± 88.31 | 231.63 ± 78.83 | 199.09 ± 76.57 | *** |
| Valine(μg/g) | 312.38 ± 61.61 | 338.31 ± 51.57 | 279.92 ± 49.68 | 284.55 ± 62.33 | 283.31 ± 53.46 | 256.88 ± 46.05 | 336.29 ± 52.25 | 362.10 ± 65.89 | 270.82 ± 41.45 | 234.41 ± 58.35 | 260.15 ± 42.96 | 219.27 ± 32.24 | 229.66 ± 35.81 | *** |
| Cystine(μg/g) | 84.15 ± 10.92 | 86.96 ± 7.24 | 83.54 ± 6.21 | 89.53 ± 8.61 | 94.08 ± 9.25 | 95.77 ± 6.99 | 93.49 ± 8.82 | 85.91 ± 7.36 | 77.87 ± 5.45 | 95.53 ± 8.28 | 90.07 ± 6.39 | 85.55 ± 5.94 | 89.57 ± 6.40 | *** |
| Methionine(μg/g) | 13.19 ± 2.87 | 14.38 ± 3.25 | 11.29 ± 3.01 | 11.56 ± 2.95 | 12.15 ± 2.88 | 12.48 ± 2.95 | 13.23 ± 2.52 | 15.18 ± 3.17 | 9.28 ± 1.49 | 16.94 ± 2.28 | 16.68 ± 2.22 | 10.25 ± 2.20 | 11.17 ± 2.21 | *** |
| Isoleucine(μg/g) | 9.66 ± 1.93 | 9.93 ± 1.59 | 8.16 ± 1.34 | 8.02 ± 1.99 | 10.36 ± 2.43 | 9.24 ± 2.01 | 12.61 ± 2.10 | 14.86 ± 2.79 | 9.90 ± 1.89 | 10.65 ± 2.08 | 9.89 ± 1.30 | 6.50 ± 1.71 | 8.31 ± 1.45 | *** |
| Leucine(μg/g) | 16.13 ± 3.30 | 16.90 ± 2.85 | 15.47 ± 3.06 | 15.91 ± 2.98 | 21.54 ± 5.22 | 18.22 ± 2.79 | 17.48 ± 2.87 | 24.47 ± 3.62 | 11.79 ± 2.75 | 20.16 ± 3.09 | 21.49 ± 2.72 | 16.59 ± 2.35 | 17.00 ± 2.12 | *** |
| Tyrosine(μg/g) | 60.92 ± 15.56 | 65.71 ± 11.75 | 53.90 ± 10.54 | 49.39 ± 12.83 | 62.00 ± 14.67 | 47.56 ± 8.99 | 41.82 ± 9.30 | 75.78 ± 10.96 | 28.87 ± 9.44 | 58.06 ± 11.85 | 70.92 ± 9.14 | 46.72 ± 10.94 | 52.21 ± 9.34 | *** |
| Phenylalanine(μg/g) | 146.33 ± 50.92 | 157.45 ± 50.91 | 103.41 ± 45.19 | 109.01 ± 57.22 | 223.34 ± 90.07 | 142.66 ± 39.53 | 132.76 ± 38.14 | 254.70 ± 49.05 | 67.74 ± 31.00 | 165.31 ± 60.49 | 200.80 ± 48.98 | 101.74 ± 43.78 | 101.10 ± 36.91 | *** |
| 4-Aminobutyric acid (GABA)(μg/g) | 100.10 ± 46.22 | 123.92 ± 44.23 | 83.54 ± 42.81 | 88.28 ± 49.97 | 140.19 ± 63.79 | 108.15 ± 41.63 | 88.15 ± 34.68 | 164.70 ± 56.58 | 39.81 ± 17.12 | 140.79 ± 57.00 | 158.91 ± 46.32 | 72.79 ± 33.65 | 68.02 ± 31.51 | *** |
| Lysine(μg/g) | 26.07 ± 13.84 | 33.16 ± 14.37 | 19.50 ± 12.08 | 18.15 ± 13.31 | 36.52 ± 19.80 | 20.09 ± 9.63 | 14.20 ± 6.01 | 47.65 ± 13.82 | 10.00 ± 3.58 | 27.44 ± 16.05 | 36.56 ± 12.72 | 13.22 ± 7.95 | 13.25 ± 6.55 | *** |
| Histidine(μg/g) | 111.52 ± 51.86 | 131.22 ± 50.33 | 86.95 ± 45.86 | 80.01 ± 50.09 | 163.89 ± 75.42 | 89.88 ± 37.21 | 59.89 ± 27.45 | 164.85 ± 46.73 | 37.81 ± 17.83 | 104.32 ± 54.14 | 154.81 ± 46.59 | 75.33 ± 38.02 | 64.84 ± 32.11 | *** |
| Tryptophan(μg/g) | 114.28 ± 58.65 | 136.31 ± 51.96 | 93.29 ± 49.26 | 88.60 ± 55.30 | 132.09 ± 64.02 | 85.32 ± 39.23 | 55.01 ± 28.48 | 141.43 ± 59.17 | 34.52 ± 13.96 | 83.45 ± 54.90 | 141.22 ± 49.28 | 71.54 ± 40.21 | 64.85 ± 32.56 | *** |
| Arginine(μg/g) | 36.22 ± 11.32 | 41.84 ± 12.16 | 31.13 ± 11.19 | 29.24 ± 12.17 | 45.93 ± 15.82 | 31.68 ± 9.31 | 25.84 ± 7.44 | 49.90 ± 11.68 | 16.52 ± 6.15 | 41.23 ± 13.77 | 47.59 ± 10.13 | 23.21 ± 9.15 | 23.21 ± 8.37 | *** |
| Proline(μg/g) | 7875.98 ± 2671.54 | 8617.34 ± 2262.90 | 6688.22 ± 2158.81 | 5937.64 ± 2080.09 | 7710.08 ± 2684.20 | 5265.17 ± 1468.14 | 3586.99 ± 1178.12 | 6913.47 ± 1908.19 | 3514.66 ± 1145.98 | 3174.83 ± 1913.41 | 5044.72 ± 1565.00 | 3590.15 ± 1337.04 | 2856.99 ± 1111.62 | *** |
| Glu-An(μg/g) | 240.65 ± 105.14 | 291.44 ± 112.78 | 182.93 ± 101.97 | 189.30 ± 114.14 | 282.26 ± 121.68 | 224.02 ± 97.41 | 197.07 ± 76.16 | 357.25 ± 76.64 | 77.81 ± 50.90 | 256.91 ± 105.28 | 392.23 ± 89.46 | 185.49 ± 81.87 | 158.85 ± 75.88 | *** |
| Fru-Amb(μg/g) | 2323.13 ± 423.66 | 2458.82 ± 283.73 | 2207.41 ± 264.83 | 2210.11 ± 330.68 | 1991.31 ± 328.34 | 2043.46 ± 299.83 | 2297.63 ± 303.57 | 2189.92 ± 319.76 | 2103.88 ± 233.47 | 1629.76 ± 207.60 | 1694.78 ± 201.92 | 1622.66 ± 161.80 | 1706.48 ± 227.45 | *** |
| Fru-His(μg/g) | 80.76 ± 36.07 | 85.78 ± 31.76 | 67.92 ± 29.23 | 55.25 ± 28.98 | 80.29 ± 28.08 | 37.96 ± 21.98 | 31.52 ± 19.83 | 68.64 ± 29.57 | 63.46 ± 18.86 | 28.91 ± 23.43 | 45.71 ± 22.08 | 32.29 ± 17.53 | 24.02 ± 14.66 | *** |
| Fru-Pro(μg/g) | 9223.37 ± 1697.31 | 9223.37 ± 1336.34 | 9223.37 ± 1377.59 | 9223.37 ± 1578.30 | 9223.37 ± 2129.71 | 8874.77 ± 1733.22 | 8951.76 ± 1965.62 | 8738.70 ± 1327.05 | 9223.37 ± 1185.44 | 4790.33 ± 2084.25 | 6291.02 ± 1039.45 | 7425.29 ± 1132.19 | 7318.31 ± 1301.53 | *** |
| Fru-Val(μg/g) | 208.71 ± 28.57 | 228.46 ± 34.09 | 205.45 ± 29.52 | 204.78 ± 32.47 | 174.12 ± 36.62 | 200.30 ± 47.08 | 264.36 ± 49.59 | 201.74 ± 46.89 | 232.15 ± 36.01 | 140.01 ± 21.55 | 174.05 ± 26.92 | 164.14 ± 29.93 | 194.44 ± 39.54 | *** |
| Fru-Thr(μg/g) | 17.42 ± 4.45 | 17.89 ± 3.68 | 15.13 ± 3.26 | 13.28 ± 2.96 | 18.42 ± 4.00 | 11.82 ± 2.67 | 12.55 ± 2.74 | 20.49 ± 4.09 | 16.82 ± 2.55 | 11.00 ± 1.93 | 12.99 ± 2.53 | 11.70 ± 2.02 | 12.12 ± 2.33 | *** |
| Fru-Gly(μg/g) | 27.94 ± 3.80 | 28.36 ± 4.25 | 23.92 ± 2.96 | 27.15 ± 3.79 | 23.30 ± 3.05 | 29.22 ± 2.75 | 32.57 ± 3.45 | 28.34 ± 3.70 | 20.62 ± 1.18 | 29.31 ± 3.37 | 30.10 ± 2.98 | 27.25 ± 2.60 | 29.61 ± 2.92 | *** |
| Fru-Ala(μg/g) | 2455.04 ± 238.34 | 2495.40 ± 164.72 | 2415.42 ± 152.37 | 2491.24 ± 245.80 | 1877.69 ± 369.84 | 2209.43 ± 233.75 | 2517.16 ± 288.46 | 2044.37 ± 242.55 | 2486.68 ± 149.38 | 1881.22 ± 261.87 | 1906.53 ± 134.67 | 2038.02 ± 168.43 | 2098.05 ± 199.14 | *** |
| Fru-Asn(μg/g) | 4267.34 ± 870.88 | 4322.83 ± 847.97 | 3523.59 ± 635.72 | 3149.42 ± 845.64 | 4368.55 ± 846.53 | 2666.50 ± 550.95 | 2886.06 ± 471.51 | 4048.61 ± 642.60 | 2954.77 ± 541.95 | 3292.33 ± 961.02 | 3321.28 ± 557.40 | 2784.13 ± 617.59 | 2836.78 ± 568.90 | *** |
| Fru-Asp(μg/g) | 1433.47 ± 220.07 | 1406.79 ± 174.64 | 1364.62 ± 160.62 | 1229.84 ± 243.84 | 1045.01 ± 230.74 | 1081.31 ± 178.23 | 1136.78 ± 196.74 | 1250.51 ± 166.68 | 1038.09 ± 215.79 | 1276.74 ± 142.40 | 1269.89 ± 158.37 | 1240.92 ± 198.85 | 1476.87 ± 163.84 | *** |
| Fru-Gln(μg/g) | 1009.79 ± 698.58 | 1208.83 ± 589.65 | 868.32 ± 604.40 | 618.88 ± 567.12 | 1343.27 ± 676.37 | 292.32 ± 316.52 | 282.84 ± 319.24 | 1242.83 ± 698.63 | 1132.88 ± 496.37 | 346.41 ± 556.75 | 507.08 ± 421.19 | 462.28 ± 416.90 | 201.50 ± 227.37 | *** |
| Fru-Glu(μg/g) | 585.32 ± 297.65 | 615.00 ± 260.75 | 521.09 ± 253.71 | 362.09 ± 231.89 | 612.80 ± 225.98 | 208.45 ± 187.84 | 199.44 ± 149.23 | 608.35 ± 238.89 | 522.83 ± 167.15 | 294.05 ± 198.46 | 404.48 ± 211.81 | 319.05 ± 172.19 | 267.96 ± 166.54 | *** |
| Fru-Ile(μg/g) | 22.12 ± 3.48 | 23.44 ± 3.90 | 21.77 ± 3.28 | 21.34 ± 3.11 | 20.58 ± 3.27 | 21.47 ± 4.20 | 31.17 ± 4.64 | 25.01 ± 3.39 | 26.07 ± 4.86 | 20.36 ± 1.91 | 24.73 ± 1.95 | 22.62 ± 2.93 | 27.93 ± 3.41 | *** |
| Fru-Leu(μg/g) | 49.44 ± 5.66 | 50.92 ± 6.24 | 44.06 ± 5.23 | 44.54 ± 6.08 | 46.77 ± 7.73 | 47.62 ± 8.34 | 64.45 ± 9.34 | 54.14 ± 8.31 | 56.99 ± 7.95 | 41.34 ± 4.85 | 46.41 ± 5.58 | 40.58 ± 5.31 | 46.87 ± 7.03 | *** |
| Fru-Tyr(μg/g) | 81.70 ± 19.02 | 81.80 ± 14.32 | 75.64 ± 13.67 | 62.57 ± 10.43 | 79.33 ± 15.95 | 54.97 ± 8.39 | 55.92 ± 10.15 | 99.62 ± 18.42 | 77.26 ± 12.13 | 51.29 ± 3.73 | 54.07 ± 6.73 | 52.31 ± 5.63 | 53.56 ± 6.05 | *** |
| Fru-Phe(μg/g) | 699.99 ± 147.82 | 744.32 ± 133.42 | 606.45 ± 138.18 | 569.09 ± 144.32 | 853.17 ± 178.15 | 579.64 ± 125.14 | 690.03 ± 166.60 | 904.79 ± 200.09 | 755.76 ± 128.10 | 385.97 ± 116.30 | 491.10 ± 133.00 | 362.38 ± 83.53 | 368.84 ± 88.95 | *** |
| Fru-Trp(μg/g) | 434.30 ± 189.07 | 479.44 ± 158.28 | 386.06 ± 153.43 | 350.67 ± 163.16 | 373.11 ± 138.81 | 260.77 ± 129.88 | 236.52 ± 106.35 | 341.58 ± 158.27 | 229.38 ± 92.59 | 180.15 ± 109.52 | 290.36 ± 120.87 | 199.29 ± 99.63 | 175.01 ± 95.30 | *** |
| Neo-phytene(mg/g) | 0.84 ± 0.09 | 0.84 ± 0.09 | 0.87 ± 0.09 | 1.00 ± 0.11 | 0.74 ± 0.11 | 1.10 ± 0.10 | 1.00 ± 0.13 | 0.71 ± 0.07 | 0.44 ± 0.06 | 1.09 ± 0.17 | 1.13 ± 0.09 | 0.88 ± 0.10 | 0.85 ± 0.09 | *** |

p-value of one-way ANOVA: ns: p-value > 0.05, *:0.05 > p-value > 0.01, **: 0.01 > p-value > 0.001, ***: p-value ≤ 0.001.
